# Characteristics of blood–brain barrier heterogeneity between brain regions revealed by profiling vascular and perivascular cells

**DOI:** 10.1038/s41593-024-01743-y

**Published:** 2024-08-29

**Authors:** Sarah J. Pfau, Urs H. Langen, Theodore M. Fisher, Indumathi Prakash, Faheem Nagpurwala, Ricardo A. Lozoya, Wei-Chung Allen Lee, Zhuhao Wu, Chenghua Gu

**Affiliations:** 1 Howard Hughes Medical Institute, Department of Neurobiology, Harvard Medical School, Boston, MA USA; 2 F.M. Kirby Neurobiology Center, Boston Children’s Hospital and Department of Neurobiology, Harvard Medical School, Boston, MA USA; 3 Helen and Robert Appel Alzheimer’s Disease Research Institute, Feil Family Brain and Mind Research Institute, Weill Cornell Medicine, New York, NY USA; 4 Present Address: Roche Pharma Research and Early Development, Neuroscience and Rare Diseases Discovery and Translational Area, Roche Innovation Center Basel, Basel, Switzerland

**Keywords:** Blood-brain barrier, Neuro-vascular interactions

## Abstract

The blood–brain barrier (BBB) protects the brain and maintains neuronal homeostasis. BBB properties can vary between brain regions to support regional functions, yet how BBB heterogeneity occurs is poorly understood. Here, we used single-cell and spatial transcriptomics to compare the mouse median eminence, one of the circumventricular organs that has naturally leaky blood vessels, with the cortex. We identified hundreds of molecular differences in endothelial cells (ECs) and perivascular cells, including astrocytes, pericytes and fibroblasts. Using electron microscopy and an aqueous-based tissue-clearing method, we revealed distinct anatomical specializations and interaction patterns of ECs and perivascular cells in these regions. Finally, we identified candidate regionally enriched EC–perivascular cell ligand–receptor pairs. Our results indicate that both molecular specializations in ECs and unique EC–perivascular cell interactions contribute to BBB functional heterogeneity. This platform can be used to investigate BBB heterogeneity in other regions and may facilitate the development of central nervous system region-specific therapeutics.

## Abstract (web summary)

Comprehensive profiling of a circumventricular organ with leaky blood vessels, and comparison to cortex vasculature reveal that blood–brain barrier heterogeneity reflects differences in endothelial cells and their interactions with perivascular cells.

## Main

The BBB is a physiological barrier between the blood and brain. Although BBB breakdown is involved in neurodegenerative diseases, an intact BBB is a major obstacle for central nervous system (CNS) drug delivery to treat neurological disorders^1^. Understanding the molecular mechanisms of BBB regulation will permit BBB manipulation for barrier repair or CNS drug delivery to improve disease treatment.

Different brain regions show different levels of blood vessel permeability. For example, the circumventricular organs (CVOs), specialized regions that include the median eminence (ME), are naturally leaky despite being adjacent to regions with a sealed BBB^2^. CVO neurons sense signaling compounds and secrete hormones into circulation to facilitate rapid communication with the periphery and regulate processes like feeding, cardiovascular function and thirst^3,4^. Moreover, BBB heterogeneity is observed in the hippocampus, basal ganglia and cerebellum; increased BBB permeability was reported in human aging and the early onset of neurodegenerative diseases^1,5,6^. Yet how these variations in BBB permeability occur is incompletely understood.

CNS capillary endothelial cells (cECs) constitute the BBB and have features like specialized tight junctions and low rates of transcytosis to regulate paracellular and transcellular trafficking, respectively^7–10^. To date, several studies have compared cECs from the CNS and peripheral tissue to identify molecular determinants of the BBB^11^. Yet BBB properties also require active induction and maintenance from the local environment^12^. Specifically, perivascular pericytes and astrocyte endfeet ensheath brain capillaries, forming the interface between ECs and neurons. Indeed, mice with reduced numbers of pericytes and astrocytes have a leaky BBB^13–16^. However, how local cues and cell interactions in the vascular microenvironment regulate regional brain barrier properties is largely unknown.

The major technical challenge to determining the mechanism underlying BBB heterogeneity is that ECs are rare in the brain, representing 4–6% of brain cells^17^. Some perivascular cells, including pericytes, are even less abundant^18^. Therefore, although typical unbiased single-cell transcriptomic studies of the brain often include vascular cells, they yield limited data about their transcriptomes owing to their relative scarcity following dissociation protocols optimized for neurons. To circumvent this problem, most studies of brain vascular and perivascular cells have relied on cell sorting from the entire brain^19,20^. This approach is not optimal for capturing BBB heterogeneity because it underrepresents smaller brain regions, which may contain transcriptionally diverse and specialized cells. Therefore, an investigation of regional vascular and perivascular cell heterogeneity necessitated the development of methods to enrich for brain ECs to discern differences in BBB-associated cells in small regions.

Here, we develop a platform to investigate how vascular and perivascular cells affect BBB functional heterogeneity in small, defined brain regions. We perform unbiased single-cell RNA sequencing (scRNA-seq) of a CVO, the ME, and a size-matched region (~0.05 × 0.2 × 1.2 mm^3^) of the somatosensory cortex (cortex) in the mouse brain. Comparison of these two small brain regions with distinct barrier properties revealed molecular differences in cECs and perivascular astrocytes and fibroblasts. Using spatial transcriptomics, we also identified molecular differences in pericytes. Correspondingly, we observed morphological differences in these cells and their interactions by electron microscopy and three-dimensional whole-brain imaging following tissue clearing by U.Clear. Finally, bioinformatics analysis identified regionally enriched ligand–receptor pairs, which may mediate the unique EC–perivascular cell interactions in these regions. Together, this work reveals both regional specializations of cECs and their unique interactions with surrounding perivascular cells, highlighting the importance of considering regional vascular and perivascular cell diversity to understand BBB heterogeneity and develop region-specific therapies.

## Results

### U.Clear reveals vascular differences in cortex and ME

**Fig. 1:**
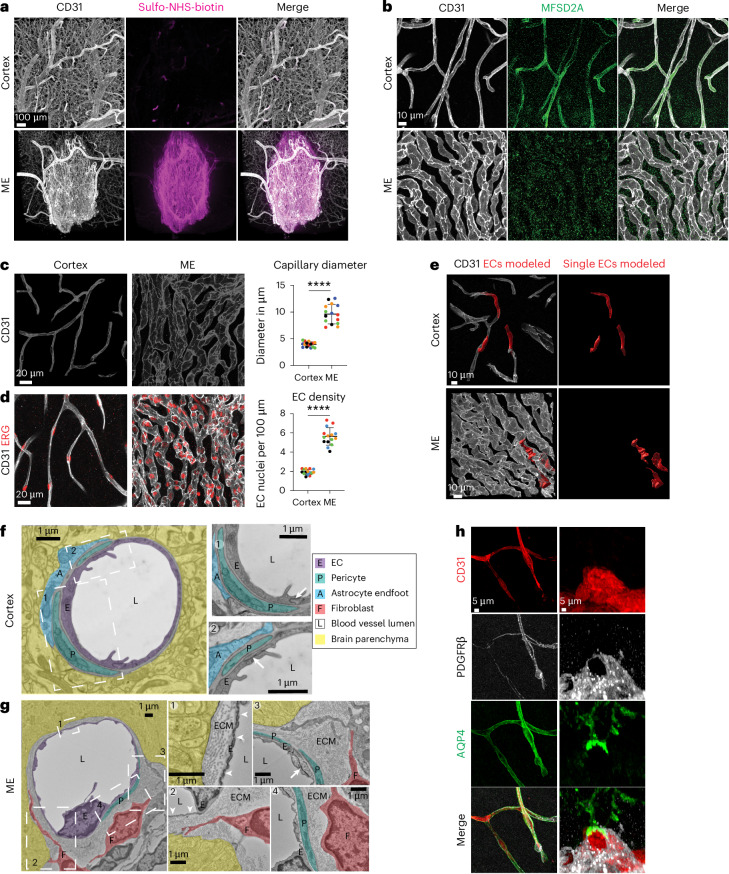
Morphological and functional differences of the vasculature between the ME and cortex. **a**, Tracer leakage assay with tracer sulfo-NHS-biotin (magenta) and immunostaining for blood vessels (CD31, white) in cortex (upper panel) and ME (lower panel) following U.Clear. Tracer in circulation was washed out by perfusion before analysis. **b**, Co-immunostaining of CD31 (white) and MFSD2A (green) in cortex and ME. **c**, High-magnification images of capillaries (CD31) highlighting vessel morphology in cortex and ME (left) and quantification of vessel diameter (right) (*n* = 5 mice, three images per region in each mouse, with the same colors showing points from the same mice). Data presented as mean ± s.d., *P* = 3.604601 × 10^−6^, nested two-tailed *t*-test. **d**, High-magnification images of capillaries (CD31, white) and EC nuclei (ERG, red) in the cortex and ME (left). Quantification shows EC density, number of endothelial cell nuclei (ERG^+^) over the length of capillaries (*n* = 5 mice, three images per region in each mouse, with the same colors showing points from the same mice). Data presented as mean ± s.d., *P* = 4.569415 × 10^−6^, nested two-tailed *t*-test. **e**, Immunostaining and 3D reconstruction of three single Tomato^+^ ECs (red) within capillaries (CD31, white) in cortex and ME. Single ECs were labeled by a single low-dose injection of 4OH-tamoxifen in adult *Cdh5*-CreER:Ai14 mice 1 week before analysis. **f**, TEM images of a cortex capillary. Pseudocolors highlight different cells: cEC (E), pericyte (P), astrocyte endfoot (A), lumen (L) and neuropil. Insets show cEC tight junctions (white arrows), pericyte cells and astrocyte endfeet. **g**, TEM images of an ME blood vessel. Pseudocolors (as in **f**) highlight different cells: cECs, pericyte, fibroblast, lumen and neuropil. Insets show capillary fenestrations (white arrowheads), cEC tight junctions (white arrows), extracellular matrix-filled perivascular space (ECM), pericyte cells and fibroblast cells. **h**, Immunostaining for CD31 (red), mural cell marker PDGFRβ (white) and astrocyte endfoot marker aquaporin 4 (AQP4, green) in cortex (left) and ME (right).

**Extended Data Fig. 1:**
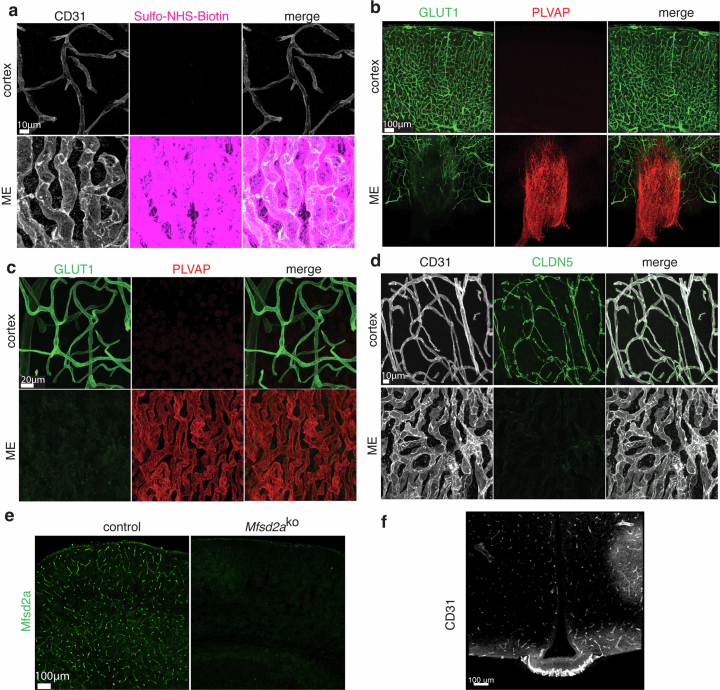
Morphological, molecular and functional differences of the vasculature between the ME and cortex. (**a**) Tracer leakage assay with Sulfo-NHS-Biotin (magenta) and immunostaining for blood vessels (CD31, white) in cortex (upper panel) and ME (lower panel). Tracer in circulation was washed out by perfusion prior to analysis. Scale bar 10 µm. (**b**) Co-immunostaining of GLUT1 (green) and PLVAP (red) in ME and cortex. Scale bar 100 µm. (**c**) High magnification images of capillaries showing distinct Glut1 (green) and Plvap (red) expression pattern and vessel morphology in the cortex and ME. Scale bar 20 µm. (**d**) Co-immunostaining of CD31 (white) and tight junction protein Cldn5 (green) in cortex and ME. Scale bar 10 µm. (**e**) Validation of specificity of newly generated polyclonal antibody against MFSD2A (green) by immunostaining of cortex from wild type and *Mfsd2a*^ko^ mice. Scale bar 100 µm. (**f**) Immunostaining of CD31 (white) in the ME in thick tissue section. Scale bar 100 µm.

We used U.Clear, an aqueous-based tissue-clearing protocol, to characterize ME and cortex blood vessels (Fig. 1a–e and Extended Data Fig. 1). U.Clear preserves endogenous fluorescence, permits the use of most antibodies to stain intact mouse tissues in their entirety and allows conventional confocal microscopy imaging. Consistent with previous reports in tissue sections^21^, after intravenous tracer injection, we observed tracer leak into the ME, but tracer remained confined to vessels in adjacent BBB-containing regions and the cortex in 3D (Fig. 1a, Extended Data Fig. 1a and Supplementary Video 1). As expected^22,23^, the ME vasculature lacks BBB markers glucose transporter 1 (GLUT1, encoded by *Slc2a1*) and claudin-5 (CLDN5) and expresses plasmalemma vesicle-associated protein (PLVAP) (Extended Data Fig. 1b–d). In addition, we found that the key BBB regulator, MFSD2 lysolipid transporter A (MFSD2A), was absent in the ME vasculature (Fig. 1b and Extended Data Fig. 1e).

Finally, with U.Clear, we observed distinct blood vessel morphologies in these regions. ME capillaries have a larger diameter and higher cEC density than cortex capillaries (Fig. 1c,d and Supplementary Video 2). Three-dimensional modeling after sparse labeling with an EC reporter (*Cdh5*-CreER; Ai14) revealed that ME capillary lumens are formed by more than one EC, while cortex capillary lumens comprised a single EC (Fig. 1e and Supplementary Video 3).

### Vascular and perivascular cell organization in cortex and ME

**Extended Data Fig. 2:**
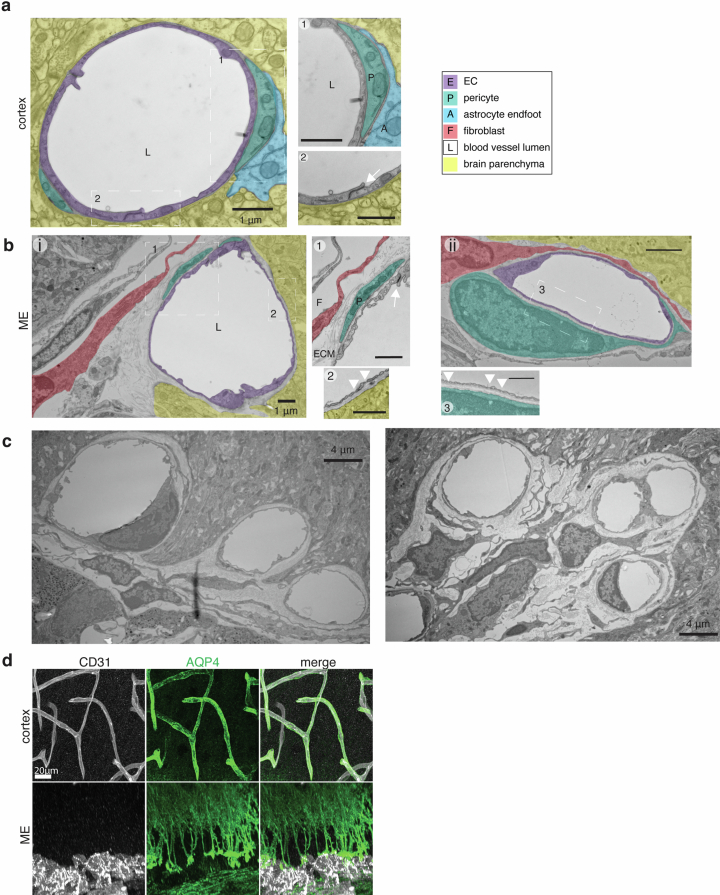
TEM reveals differences in organization of the vasculature and cellular environment in the ME and cortex. (**a**) TEM images of a cortical capillary. As outlined in the legend, pseudocolors highlight different cells: cEC (purple), pericyte (teal), astrocyte endfoot (cyan), lumen (L, white) and neuropil (yellow). Insets show cEC tight junctions (white arrows), pericyte cells (P, teal) and astrocyte endfeet (A, cyan). Scale bar represents 1 µm (left). (**b**) TEM images of two blood vessels in the ME, (i) and (ii). As outlined in the legend, pseudocolors highlight different cells: cEC (purple), pericyte (teal), fibroblast (red), lumen (L, white) and neuropil (yellow). Insets show capillary fenestrations (white arrowheads), cEC tight junctions (white arrows), extracellular matrix-filled perivascular space (ECM), pericyte cells (P, teal) and fibroblast cells (F, red). Scale bar represents 1 µm. (**c**) TEM images of two groups of ME blood vessels. Scale bar represents 4 µm. (**d**) Co-immunostaining for astrocyte endfoot marker Aqp4 (green) and CD31 (white) in cortex and ME. Scale bar 20 µm.

To understand how vascular and perivascular cells contribute to functional differences in BBB permeability, we first used transmission electron microscopy (TEM) to examine their interactions at the ultrastructural level. Cortex cECs are well known to interact closely with pericytes and astrocyte endfeet (Fig. 1f and Extended Data Fig. 2a). However, studies from several species indicated that ME perivascular cell interactions are quite different^24^. Indeed, we found that ME cECs are fenestrated and share a basement membrane with pericytes. Surprisingly, we did not see typical astrocyte endfeet surrounding ME capillaries. Rather, fibroblasts were present in a large perivascular space filled with extracellular matrix (Fig. 1g and Extended Data Fig. 2b,c). Tanycytes, specialized glial cells in CVOs^2^, were also not readily distinguishable in the ME parenchyma, which abuts the ME perivascular space on the dorsal side.

U.Clear revealed that cortex cECs, pericytes and astrocyte endfeet interact closely, and ME pericytes and fibroblasts (platelet-derived growth factor receptor beta (PDGFRβ)^+^) interact with cECs in the perivascular space (Fig. 1h). However, AQP4 is not polarized at ME astrocyte endfeet but rather is found throughout processes extending toward the ME vasculature (Extended Data Fig. 2d). These notable structural differences motivated us to identify their molecular underpinnings.

### Regionally enriched cell types in cortex and ME by scRNA-seq

**Fig. 2:**
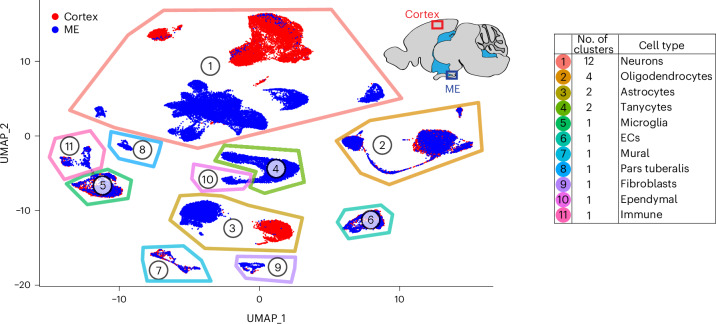
ME and cortex cell types profiled by scRNA-seq. **a**, Uniform manifold approximation and projection (UMAP) plot of 58,238 single-cell transcriptomes (35,934 from ME and 22,304 from cortex). Cell-type clusters were annotated post hoc based on their transcriptional profiles (Methods). The number of clusters identified for each cell type is indicated in the plot legend.

**Extended Data Fig. 3:**
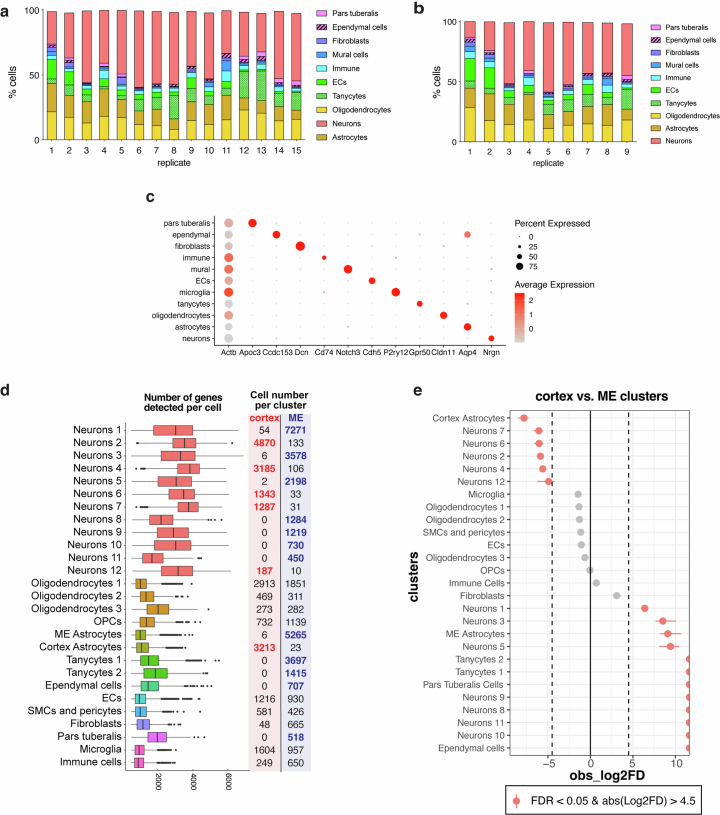
Single cell RNA sequencing of median eminence and a size-matched region of somatosensory cortex reveals unique cell types in each brain region. (**a**) Bar plot showing distribution of cells in each cell type for each experimental replicate. Replicates 12–15, in bold, are from the ME region only. (**b**) Bar plot showing distribution of cells in each cell type for each library batch preparation. Batch 9, highlighted in bold, is comprised of libraries from the ME region only. (**c**) Dot plot showing average expression of one cell type-specific transcript used to annotate cluster cell types in Fig. 2. Additional transcripts used for annotation are detailed in Methods. (**d**) Tukey box and whisker plot depicting the number of genes detected per cell in all identified clusters in Fig. 2. Box shows the median and first and third quartiles, whiskers represent 1.5 times the interquartile range. The cell number in each cluster per sample region is indicated at the right of each plot, with cortex-enriched clusters highlighted in red and ME-enriched clusters highlighted in blue (as determined in (**e**)). Data was collected on 15 separate days, with two technical replicates from ME and cortex samples in each replicate (except for replicates 12–15, which were ME only). ME and cortex regions were isolated from the same 5 mice in each replicate and pooled by region prior to dissociation. (**e**) Point-range plot showing the relative differences in cell proportions for each cluster in (**d**) between the ME and cortex. Regional enrichment was determined by permutation test, with significance assessed by false discovery rate following bootstrapping (implemented by the scProportionTest R package). Clusters showing regional enrichment (log_2_ fold-change greater than |4.5| and FDR < 0.05) are labeled and shown in red.

To identify molecular differences in ECs and perivascular cells, we performed inDrops scRNA-seq^25,26^ of the ME and a size-matched region of the cortex. We developed a tissue dissociation protocol to obtain efficient, unbiased recovery of vascular cells. All blood vessel-associated cell types are well represented in our dataset, with ECs comprising ~4% of cells, on par with estimates of their prevalence in the mouse brain. After quality control filtering (Methods), 58,117 high-quality cells were retained for further analysis; 35,879 from ME and 22,238 from the cortex. Unbiased cell clustering with Seurat identified 27 clusters corresponding to 11 cell types based on the expression of cell-type-specific transcripts (Fig. 2, Extended Data Fig. 3, Supplementary Figs. 1–3 and Supplementary Table 1). Notably, astrocytes from the ME and cortex clustered separately (Fig. 2 and Extended Data Fig. 3d,e).

**Fig. 3:**
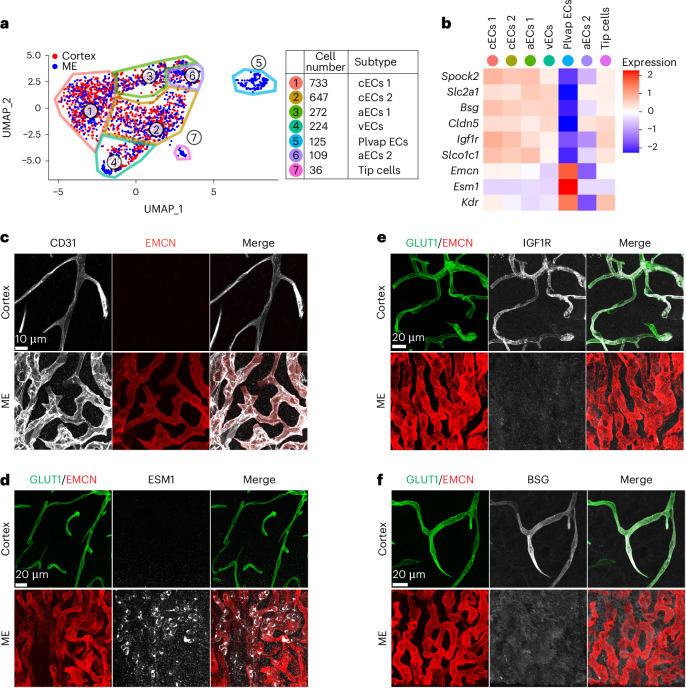
ME contains region-specific cECs. **a**, UMAP plot of 2,284 EC transcriptomes. Seven subtypes of ECs were identified with an unbiased analysis based on their transcriptional profiles (see Extended Data Fig. 4a). The number of each cell subtype profiled is indicated in the plot legend. **b**, Heatmap illustrating the average relative expression of regionally enriched genes in each subtype cluster identified in **a** that were validated by immunostaining. Regionally enriched genes show an average log_2_(fold change) > 0.6 (>1.5-fold change in expression) with an adjusted *P* < 0.05 by two-sided Wilcoxon test. **c**, Co-immunostaining for ME cEC-enriched EMCN (red) with CD31 (white) in cortex and ME. **d**, Co-immunostaining for ME cEC-enriched ESM1 (white), EMCN (red) and cortex cEC-enriched protein GLUT1 (green) in cortex and ME. **e**, Co-immunostaining for cortex cEC-enriched IGF1R (white), ME cEC-enriched protein EMCN (red) and cortex cEC-enriched protein GLUT1 (green) in cortex and ME. **f**, Co-immunostaining for cortex cEC-enriched BSG (white), GLUT1 (green) and EMCN (red) in cortex and ME.

**Extended Data Fig. 4:**
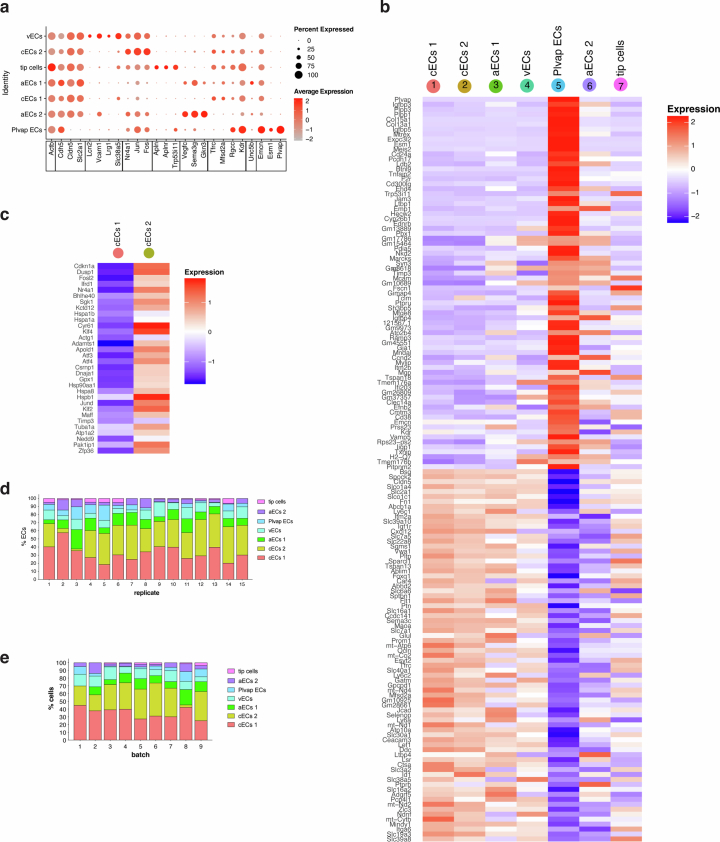
Novel regionally enriched genes identified in cECs in the ME and cortex. (**a**) Dot plot of average expression of vascular zonation markers and several transcripts used to annotate cluster cell types in Fig. 3a. *Actb* and *Cdh5* are expressed in all populations, and *Cldn5* and *Slc2a1* are cortex-enriched transcripts, as illustrated in Extended Data Fig. b-d. *Lcn2, Vcam1, Lrg1*, and *Slc38a5* are enriched in vECs; *Nr4a1, Jun* and *Fos* are enriched in cECs 2; *Apln, Aplr* and *Trp53i11* are enriched in tip cells; *Vegfc*, *Sema3g* and *Gkn3* are enriched in aECs 1 and 2; *Tfrc* and *Mfsd2a* are enriched in cECs 1 and 2; *Emcn, Esm1*, and *Plvap* are enriched in ME cECs; *Unc5b* is enriched in aECs 1; *Rgcc* and *Kdr* are enriched in capillary ECs (cECs 1 and 2 and ME cECs). (**b**) Heatmap illustrating the top 75 differentially expressed genes from each group when comparing cortex-derived cECs 1 and cECs 2 with ME cECs. Differentially expressed genes were determined by two-sided Wilcoxon test in Seurat (minimum percentage = 25%, log2-fold change > 0.6, adjusted p-value < 0.05). (**c**) Heatmap illustrating endothelial activity-induced transcripts (reported in Hrvatin, et al.^76^) that are differentially expressed between cECs 1 and cECs 2. Differentially expressed genes were determined by two-sided Wilcoxon test in Seurat (minimum percentage = 10%, log2-fold change > 0.25, adjusted p-value < 0.05). (**d**) Percentage of EC subtypes sequenced by experimental replicate. Experimental replicates with only ME samples are highlighted in blue. (**e**) Percentage of EC subtypes sequenced by library batch. Library batches with only ME samples are highlighted in blue.

**Extended Data Fig. 5:**
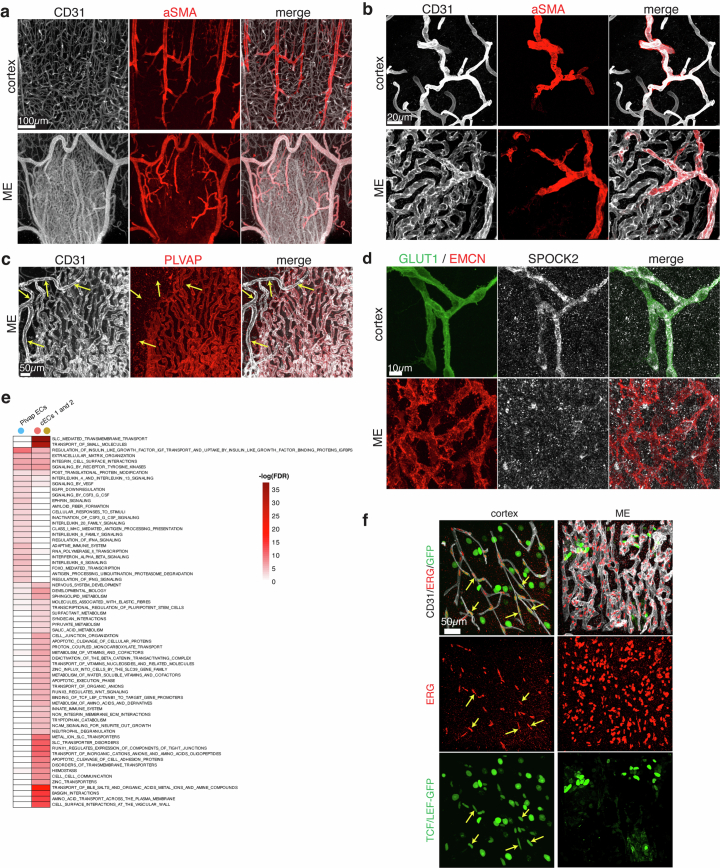
Immunostaining reveals ME capillary boundary and validates differential gene expression across regions. (**a**) Co-immunostaining with anti-CD31 (white) and anti-SMA (red) antibodies to visualize arteries in the cortex and ME. Scale bar 200 µm. (**b**) High magnification images of blood vessels (CD31, white), highlighting vessels interacting with SMA-positive smooth muscle cells (red) in cortex (top) and ME (bottom). Scale bar 20 µm. (**c**) Co-immunostaining with anti-CD31 (white) and anti-PLVAP (red) antibodies in the ME. Yellow arrows indicate arteries in this region. Scale bar 50 µm. (**d**) Co-immunostaining of SPOCK2 (white), GLUT1 (green) and EMCN (red) in the cortex and ME of P5 wild type mouse. Scale bar 10 µm. (**e**) Heatmap showing significantly upregulated Reactome pathways for the top 115 differentially expressed genes in cortex-derived cECs 1 and 2 and ME cECs. Differentially expressed genes were determined by two-sided Wilcoxon test in Seurat comparing cortex-derived cECs 1 and 2 with ME cECs (minimum percentage = 25%, log2-fold change > 0.6, adjusted p-value < 0.05). p-value was calculated with a one-sided Fisher’s exact test, and -log(FDR) values are shown. Upregulated pathways from other pathway databases can be found in Supplementary Table 1. (**f**) Co-immunostaining for CD31 (white), endothelial nuclei marker ERG (red), and GFP in cortex and ME of TCF/LEF-GFP Wnt-signaling reporter mice. GFP expression (green) indicates activation of the Wnt-signaling pathway. Arrows indicate ERG and GFP double positive nuclei. Scale bar 50 µm.

We first investigated EC regional differences by performing subclustering analysis, finding seven subtypes, including capillary ECs (cECs 1 and 2), arteriolar ECs (aECs 1 and 2) and venous ECs (vECs) (Fig. 3a, Extended Data Fig. 4 and Methods). ECs from the ME were found in all subclusters because ECs from ME-adjacent regions with BBB-containing blood vessels are inevitably included in our dissection. Despite this, an ME-specific EC subtype expressing *Plvap* emerged, which we confirmed by immunostaining (Fig. 3a, Extended Data Fig. 4a,b and Supplementary Table 1). *Plvap*-expressing ECs are cECs based on the expression of markers attributed to cECs^20^ (but not arteriolar or venous markers) and the absence of smooth muscle cells (which envelop arteries but not capillaries) (Extended Data Figs. 4a and 5a–c). Thus, we will refer to these cells as ‘ME cECs’. Finally, a small cluster of ECs was derived predominantly from the ME (36 out of 37 cells) that expressed markers characteristic of tip cells^20^, consistent with the characterization of this region as angiogenic^27^. Thus, we captured rare EC subtypes in small brain regions and ECs from all segments of the vascular tree, demonstrating that we can perform fine-grained molecular analysis and effectively investigate EC and perivascular cell heterogeneity with our method.

### ME and cortex cECs show transcriptional differences

**Fig. 4:**
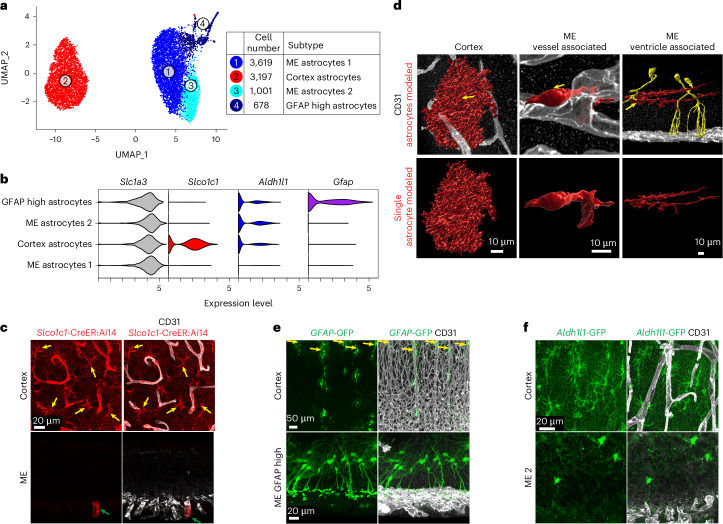
Distinct ME astrocyte subtypes observed with unique interactions with blood vessels. **a**, UMAP plot of 8,508 astrocyte transcriptomes. Astrocyte subtypes were identified with an unbiased analysis. The number of each cell subtype profiled is indicated in the plot legend. **b**, Violin plot showing the expression level of a subset of differentially expressed genes in each cluster identified in **a**. Differentially expressed genes show an average log_2_(fold change) of >0.6 (>1.5-fold change in expression) with an adjusted *P* value of <0.05 by two-sided Wilcoxon test. For comparison of ME astrocyte populations, coronal orientation is shown in each ME panel in **c**–**f**, with blood vessels on the bottom and the third ventricle toward the top. **c**, Left: Tomato in the cortex and ME of *Slco1c1*-CreER:Ai14 mice. Tomato (red) indicates *Slco1c1* expression. Right: co-staining of CD31 (blood vessels, white). Note Tomato expression in cortex capillaries and astrocytes, but not ME capillaries and astrocytes. Single Tomato^+^ vessel in ME (green arrow) is arterial. Yellow arrows point at astrocytes in the cortex. **d**, Fluorescent labeling of astrocytes in cortex and ME using *Glast*-CreER:Ai14 mice after low-dose 4OH-tamoxifen to achieve sparse cell labeling. Top row shows immunostaining for Tomato^+^ astrocytes (red) and blood vessels (CD31, white). Yellow arrow indicates the location of the cell body, as determined by DAPI staining. Bottom row displays 3D reconstructions of astrocytes (red). Cells modeled in yellow are tanycytes. For a comparison of different astrocyte populations with the same scales, see Extended Data Fig. 7b. **e**, Left: GFP (green) in the cortex and ME of GFAP-GFP mice. Right: co-staining for CD31 (white, vessels). In the cortex, GFP only sparsely labeled peri-arterial astrocytes (yellow arrows). Scale bar, 50 µm in the top row and 20 µm in the middle and lower rows. **f**, Left: fluorescent labeling (green) of cortex astrocytes and ventricle-associated ME 2 astrocytes using *Aldh1l1*-GFP mice. Right: co-staining for CD31 (white) to label capillaries.

We evaluated regional cEC differences, finding 445 differentially expressed genes (Extended Data Fig. 4b and Supplementary Table 1). We validated nine of these genes (summarized in Fig. 3b) by immunostaining: endomucin (EMCN) and endothelial cell-specific molecule 1 (ESM1) are expressed in ME cECs but not cortex cECs (Fig. 3c,d), whereas insulin-like growth factor 1 receptor (IGF1R), basigin (BSG) and SPARC/osteonectin, Cwcv and Kazal-like domains proteoglycan 2 (SPOCK2) (Fig. 3e,f and Extended Data Fig. 5d) are expressed in cortex cECs but are not detected in ME cECs (additional validation in Figs. 4f and 7d–f). These six cortex cEC-enriched genes are expressed in BBB-containing cECs throughout the brain, acting as common BBB-related genes. However, we anticipate that other cortex cEC-enriched genes may be expressed in a region-specific manner.

Gene set enrichment analysis of cEC differentially expressed genes corresponded to regional functional differences (Extended Data Fig. 5e and Supplementary Table 1). Cortex cEC genes were enriched for BBB-related pathways, including canonical Wnt signaling, in part owing to the expression of *Lef1*. We confirmed a lack of LEF1 activity in the ME^28^ (Extended Data Fig. 5f), consistent with reports of low Wnt activity in CVOs^22,23^. ME cEC-enriched genes were related to pathways like ghrelin signaling, in accordance with the role of ME in the hunger response^29^. Additionally, we observed enrichment for vascular endothelial growth factor (VEGF) signaling, in part owing to different expression patterns of VEGF receptors in ME and cortex cECs (*Vegfr2* vs *Vegfr1*; Extended Data Fig. 4b and Supplementary Table 1).

**Extended Data Fig. 6:**
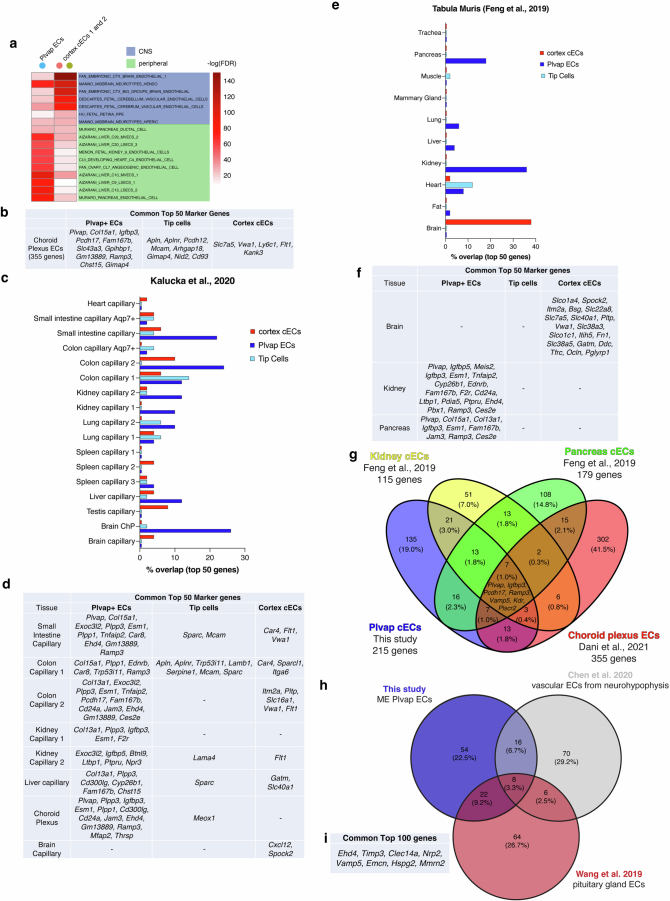
Comparison of scRNAseq of ME-derived Plvap + ECs to published ECs from the mouse neurohypophysis, pituitary gland and peripheral organs. (**a**) Heatmap showing the top 10 most similar cell types when analyzing the top 115 differentially expressed genes for each cEC subtype. Differentially expressed genes were determined by two-sided test in Seurat comparing each subcluster to all other astrocyte cell subclusters with min.pct=0.25 and logFC>0.6. Hypergeometric p-value was calculated and -log(FDR) values are shown. Brain cell types are highlighted in blue, while peripheral cell types are highlighted in green. (**b**) Table showing genes common between the top 50 differentially expressed genes in Plvap ECs, tip cells and cECs1 (calculated by two-sided Wilcoxon test in Seurat comparing each EC subtype to all other ECs with min.pct=0.25 and logFC>0.6) and marker genes of ECs from the choroid plexus from Dani et al.^31^. (**c**) Bar plot showing the percentage overlap of the top 50 enriched genes from Plvap ECs, tip cells and cECs 1 EC subtypes (calculated by two-sided Wilcoxon test in Seurat comparing each EC subtype to all other ECs with min.pct=0.25 and logFC>0.6) and each of the top 50 enriched genes in ECs reported from Kalucka et al.^20^. (**d**) Table showing genes common between Plvap+ ECs, tip cells and cECs 2 in the organs with the highest level of overlap in (c). (**e**) Bar plot showing the percentage overlap of the top 50 enriched genes from Plvap ECs, tip cells and cECs 1 EC subtypes (calculated by two-sided Wilcoxon test in Seurat comparing each EC subtype to all other ECs with min.pct=0.25 and logFC>0.6) and each of the top 50 enriched genes in ECs reported from Feng et al.^30^. (**f**) Table showing genes common between Plvap+ ECs, tip cells and cECs 1 in the organs with the highest level of overlap in (e). (**g**) Venn diagram showing the overlap between EC marker genes from the kidney and pancreas from Feng et al.^30^ and the choroid plexus from Dani et al.^31^ with marker genes of Plvap ECs (calculated by two-sided Wilcoxon test in Seurat comparing Plvap ECs to all other ECs with min.pct=0.25 and logFC>0.6). The 9 genes common to all samples are listed in the center. (**h**) The top 100 genes enriched in ME-derived Plvap+ ECs when compared to cortex-derived cECs (as in (a), blue) were compared to the top 100 genes enriched in vascular ECs from the mouse neurohypophysis^32^ (gray) and the top 100 genes enriched in ECs isolated from the mouse pituitary gland^23^ (red). Plvap+ EC enriched genes were calculated by two-sided Wilcoxon test in Seurat comparing each EC subtype to all other ECs. (**i**) Table containing the identities of the 8 genes expressed by all samples compared in (h).

Given that ME blood vessels share features with blood vessels in peripheral organs, we next compared gene expression patterns between ME or cortex cECs and cECs from peripheral tissues. We performed gene set enrichment for cell type signatures and determined the overlap of enriched genes in ME cECs, tip cells and cortex cECs with published datasets profiling cECs throughout the body^20,30,31^. Cortex cECs showed little similarity to peripheral ECs, whereas ME cECs showed overlap with ECs from the choroid plexus and the pancreas, kidney, colon and small intestine (Extended Data Fig. 6a–g), organs with fenestrated vessels. Moreover, a comparison of our data to ECs from the mouse pituitary gland^23^ and neurohypophysis^32^, which are adjacent to the ME, reveals some overlap (Extended Data Fig. 6h,i). Together, the ~400 molecular differences between cECs in the ME and cortex indicate that the differences in BBB permeability are at least in part a result of the molecular specialization of cECs.

### ME astrocyte subtypes and their association with capillaries

**Extended Data Fig. 7:**
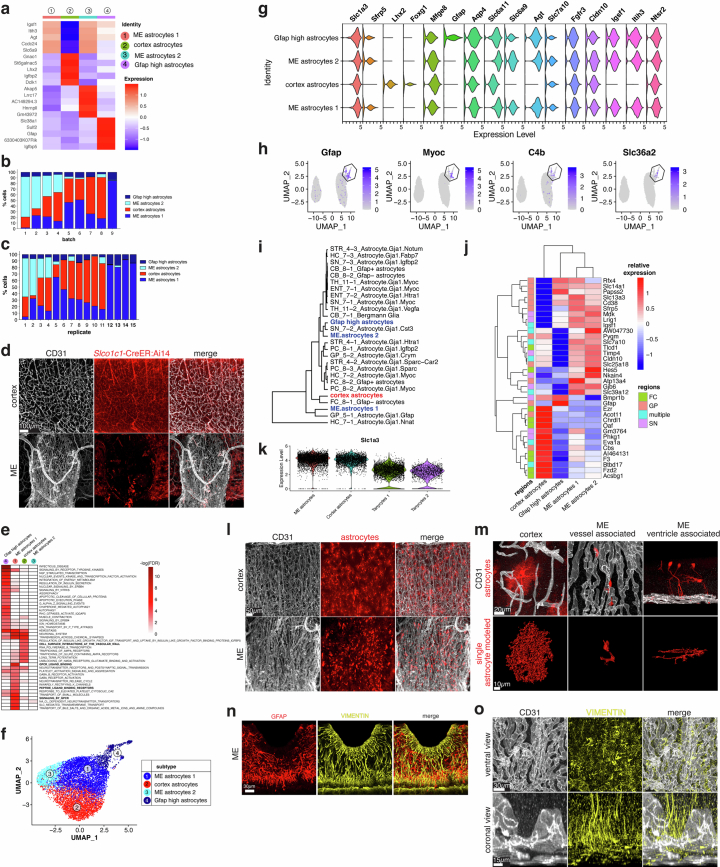
Astrocyte subtypes and their interactions with blood vessels are distinct between the ME and cortex and from published datasets. (**a**) Heatmap illustrating the top 5 genes differentiating each astrocyte cell subcluster. Differentially expressed genes were determined by two-sided Wilcoxon test in Seurat comparing each subcluster to all other astrocyte cell subclusters. (**b**) Percentage of astrocyte subtypes sequenced by experimental replicate. Experimental replicates with only ME samples are highlighted in bold (12–15). (**c**) Percentage of astrocyte subtypes sequenced by library batch. Library batches with only ME samples are highlighted in bold (9). (**d**) Fluorescent labeling of cortex blood vessels and astrocyte populations (Tomato, red) in cortex and ME using *Slco1c1*-CreER:Ai14 mice. Co-staining for blood vessels (CD31, white). Scale bar 100 µm. (**e**) Heatmap showing the significance of upregulated pathways for the top 50 differentially expressed genes for each astrocyte subtype. Differentially expressed genes were determined by two-sided Wilcoxon test in Seurat comparing each subcluster to all other astrocyte cell subclusters with min.pct=0.25 and logFC>0.6. p-value was calculated with a one-sided Fisher’s exact test, and -log(FDR) values are shown. (**f**) UMAP projections of astrocyte transcriptomes following Harmony cross-correlation analysis. (**g**) Violin plots showing the expression of GLAST (*Slc1a3*), *Sfrp5*, and of markers reported previously^35^ of telencephalon astrocytes, expressed in cortex astrocytes (*Lhx2, Foxg1, Mfge8*) and diencephalon astrocytes expressed in ME astrocytes (*Gfap, Aqp4, Slc6a11, Agt, Slc7a10, Fgfr3, Cldn10, Igsf1, Itih3, Ntsr2*). (**h**) UMAP plots highlighting the expression of markers (purple) reported previously^35^ of Myoc-expressing astrocytes in Gfap high population (outlined in black). (**i**) Clustergram showing similarity of ME and cortex astrocyte subtypes to those reported previously^36^. The expression patterns of the top 25 differentially expressed genes in the 4 astrocyte subtypes (calculated by two-sided Wilcoxon test in Seurat comparing each astrocyte subtype to all other astrocytes, with min.pct = 0.25 and logFC > 0.6) were clustered in aggregate metacells using the pheatmap R package with the default parameters. (**j**) Heatmap (scaled by row) illustrating the top 15 genes distinguishing the FC_8-1_Gfap- astrocytes (FC), GP_5-1.Astrocyte.Gja1.Gfap (GP), and SN_7-2_Astrocyte.Gja1.Cst3 (SN) subtypes previously reported^36^. Genes expressed in at least 2 of the above astrocyte subtypes are labeled as ‘multiple.’ (**k**) Violin plot showing expression of *Slc1a3*, which encodes GLAST, in astrocyte and tanycyte populations. (**l**) Fluorescent labeling of astrocyte populations (Tomato, red) in cortex and ME using Glast-CreER:Ai14 mice with a high dose of tamoxifen. Co-staining for blood vessels (CD31, white). Scale bar 50 µm. (**m**) Fluorescent labeling of astrocyte populations in the cortex and ME using Glast-CreER:Ai14 mice after a low dose of tamoxifen to achieve sparse cell labeling. Upper row: immunostaining for Tomato-positive astrocytes (red) and blood vessels (CD31, white). Lower row displays 3D reconstructions of astrocytes (red). Scale bar 20 µm (upper) and 10 µm (lower). (**n**) Co-immunostaining for GFAP-enriched astrocyte population (GFAP, red) and tanycyte marker Vimentin (yellow). Scale bar 30 µm. (**o**) Co-immunostaining for tanycyte marker VIMENTIN (yellow) and pan EC marker CD31 (white) in ME. Ventral and coronal view of ME shown, note tanycyte protrusions in touch with ME vessels. Scale bar 30 µm (upper panel) and 15um (lower panel).

Astrocyte endfeet ensheath brain capillaries with BBB properties; however, we found that ME astrocytes lack typical endfoot features (Fig. 1g,h and Extended Data Fig. 2), and astrocytes from each region clustered separately by scRNA-seq (Figs. 2 and 4a and Extended Data Fig. 7a–c). One gene, *Slco1c1*, previously known to be expressed in cortex astrocytes and cECs^33^, was absent in ME astrocytes and cECs (Figs. 3b and 4b). We validated this expression pattern with *Slco1c1-*CreER:Ai14 reporter mice (Fig. 4c and Extended Data Fig. 7d). Pathway analysis showed enrichment for ‘cell surface interactions at the vascular wall’ in cortex astrocytes and ‘GPCR signaling’ and ‘peptide-receptor interactions’ in ME astrocytes (Extended Data Fig. 7e).

Subclustering analysis identified four astrocyte subtypes, one from the cortex and three from the ME (Fig. 4a): ‘ME 1’, ‘ME 2’ and, consistent with previous reports in other CVOs, a subtype with high expression of *Gfap* predominantly (651 out of 678; 96%) from the ME (‘*Gfap* high’). Harmony analysis^34^ confirmed all subtypes are in a similar cell state (Extended Data Fig. 7f). We next compared these astrocyte subtypes to two published datasets that profiled multiple brain regions; our cortex astrocytes express markers of protoplasmic astrocytes from one of the datasets^35^ and *Gfap*-low frontal cortex astrocytes from the other^36^. Our ME astrocytes express markers of the diencephalon, and *Gfap*-high astrocytes express markers similar to ‘dorsal midbrain Myoc-expressing’ cells as reported in ref. ^35^ (Extended Data Fig. 7g,h). In ref. ^36^, ME subtypes showed similarity to substantia nigra and globus pallidus astrocytes (Extended Data Fig. 7i,j). Thus, ME astrocytes most likely represent novel subtypes.

We also found that ME and cortex astrocytes associate differently with blood vessels. We used reporters driven by *Slc1a3* (*Glast*-CreER:Ai14) or *Gfap* (GFAP-EGFP^37^) to visualize individual astrocytes. *Slc1a3* encodes GLAST and is expressed in both regions (Fig. 4b and Extended Data Fig. 7k). As expected, cortex GLAST^+^ astrocytes were stellate, with cell bodies situated away from the vasculature and extending numerous processes around blood vessels (Fig. 4d, Extended Data Fig. 71,m and Supplementary Video 4). GLAST^+^ ME astrocytes exhibited two morphologies (Fig. 4d, Extended Data Fig. 7l,m and Supplementary Video 4): one subtype was directly associated with ME blood vessels, nestled between the vessels with few, short processes, and the other subtype had cell bodies near the ventricle and long processes extending into the ME region but not associating with blood vessels. The third ME subtype, *Gfap*-EGFP^+^ astrocytes (‘*Gfap* high’) had cell bodies near the ventricle and extended numerous processes toward the vasculature (Fig. 4e, Extended Data Fig. 7n,o and Supplementary Video 4). To distinguish ME astrocyte subtypes 1 and 2, *Aldh1l1*-EGFP reporter mice were used. *Aldh1l1* is expressed by ME 2 and *Gfap*-high astrocytes (Fig. 4b). *Aldh1l1*-expressing ME astrocytes have cell bodies near the ventricle and extend few processes toward the vasculature (Fig. 4f), indicating that ME 2 astrocytes correspond to the astrocytes interacting more distantly with the ME vasculature. Therefore, it is plausible that the ME 1 subtype represents the astrocytes nestled between ME blood vessels.

Thus, ME astrocytes are molecularly distinct from cortex astrocytes, lack endfeet typical of cortex astrocytes and show limited vascular association. In line with these differences, we found that most of the top 100 differentially expressed genes between ME and cortex astrocytes are predicted to be secreted or associated with the cell membrane (64% and 70%, respectively; Supplementary Table 1), suggesting that astrocyte molecular differences may be related to EC–astrocyte signaling (elaborated in Fig. 7).

### Cortex pericyte–cEC interaction features by serial TEM

**Fig. 5:**
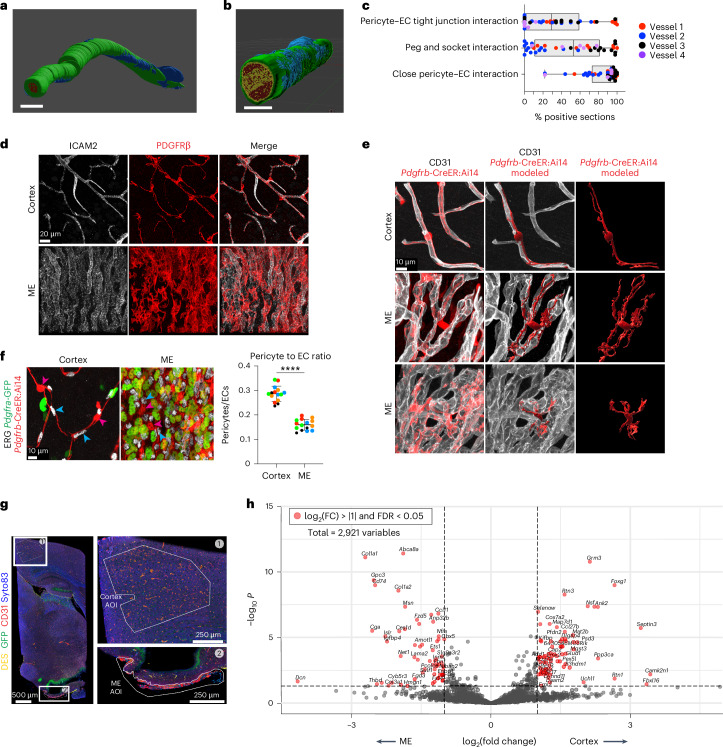
Pericytes associated with cortex and ME blood vessels show distinct molecular, morphological and anatomical features. **a**,**b**, Serial TEM reconstruction of EC and pericyte interactions in two cortex blood vessels. ECs are shown in green, pericytes in blue and the blood vessel lumen in red. Scale bars, 5 µm. **c**, Quantification of three features of pericyte–EC interaction in vessels in **a** and **b** and two additional vessels (*n* = 4 total vessels from one animal), displayed as a percentage of sections showing each feature. Each point represents a 50-section increment. Whiskers span the smallest and largest values, and the boxplot shows the median and first and third quantiles. **d**, Co-immunostaining for pan-pericyte marker PDGFRβ (red) and pan-EC marker ICAM2 (white) in cortex and ME. **e**, Immunostaining and 3D reconstruction of single Tomato^+^ pericytes (red) in touch with capillaries (CD31, white) in cortex and ME (two examples). Single pericytes labeled by single low-dose injection of 4OH-tamoxifen in adult *Pdgfrb*-CreER:Ai14 mice 1 week before analysis. **f**, Left: co-immunostaining for EC nuclei marker ERG (white) and pericytes labeled using *Pdgfrb*-CreER:Ai14; *Pdgfra*-GFP mice. Magenta arrowheads point at GFP^−^Tomato^+^ pericytes; cyan arrowheads point at ERG^+^ EC nuclei. Right: quantification of pericyte (GFP^−^Tomato^+^) to EC (ERG^+^) ratio using *Pdgfra*-H2B-GFP; *Pdgfrb*-CreER:Ai14 mice (*n* = 5 mice, for quantification three images per region and mouse were taken, same colors refer to same mice, data presented as mean ± s.d., *P* = 4.793057 × 10^−6^, nested two-tailed *t*-test). **g**, Co-immunostaining for pericyte marker desmin (DES, yellow), GFP (to visualize *Pdgfra*-H2B-EGFP in fibroblasts, green), EC marker CD31 (red) and nuclear marker Syto83 (blue) for GeoMX area of interest morphological identification. **h**, Volcano plot of differentially expressed genes between ME and cortex pericyte-enriched region of interest from GeoMX whole transcriptome profiling (also shown in Extended Data Fig. 8f). Differential expression was determined by linear mixed model analysis and significance assessed by FDR. Red points show log_2_(fold change) > |1| and FDR < 0.05 between cortex and ME pericyte-enriched regions of interest.

**Extended Data Fig. 8:**
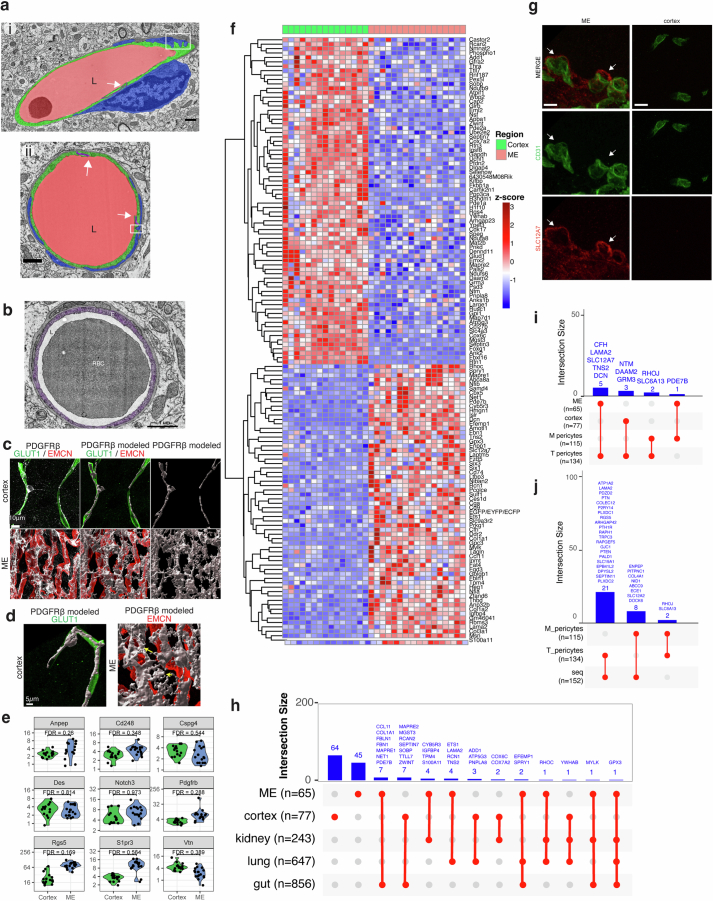
Mural cells associated with cortex and ME blood vessels show distinct morphology and transcriptomic differences. (**a**) Representative cross section images of (i) vessel 1 and (ii) vessel 2 reconstruction by serial TEM. Pseudocolors show reconstructed regions: blood vessel lumen (L, red); EC (green); and pericyte cell (blue). White arrows indicate EC tight junctions (purple), and white boxes highlight ‘peg and socket’ pericyte-EC interactions. Scale bar represents 1 µm. (**b**) Representative image of cortex vessel 4 from serial TEM dataset. Scale bar represents 1 µm. (**c**) Immunostaining for PDGFRβ (white, left panels) and Imaris 3D reconstruction of pericytes (white, middle and right panels) in cortex and ME. Co-staining for Glut1 (green) and Emcn (red) to mark capillaries in the cortex and ME, respectively. Scale bar 10 µm. (**d**) High magnification images of reconstructed pericytes (PDGFRβ, white) in contact with capillaries (Glut1, green and Emcn, red) in cortex and ME. Arrows point at ME pericyte protrusions not in contact with capillaries. Scale bar 5 µm. (**e**) Violin plots showing expression of published markers of brain pericytes in both ME and cortex areas of interest from GeoMX whole transcriptome profiling. (**f**) Heatmap of differentially expressed genes (log_2_ fold-change greater than |1| and FDR < 0.05) between cortex and ME pericyte-enriched regions of interest from GeoMX whole transcriptome profiling (also shown in Fig. 5h). Differential expression was determined by linear mixed model analysis and significance assessed by false discovery rate (FDR). (**g**) Immunostaining for CD31 (green) and SLC12A7 (red) in ME and cortex in coronal tissue sections. Scale bar 10 µm. (**h**) Upset plot showing overlap between human pericyte cell type signatures and differentially expressed genes in ME and cortex pericyte-enriched regions. (**i**) Upset plot showing overlap between human brain pericyte cell type signatures and ME and cortex pericyte-enriched differentially expressed genes (Fig. 5h). (**j**) Upset plot showing overlap between human pericyte cell type signatures and pericyte marker genes from our scRNAseq dataset (calculated by two-sided Wilcoxon test in Seurat comparing pericytes to other mural cells, with min.pct=0.25 and logFC>0.6).

Given that cEC physical interaction with pericytes is important for the BBB, we first examined cortex cEC–pericyte interactions using serial TEM. We reconstructed pericytes and cECs in two capillaries from a published mouse visual cortex dataset^38^, as we expect features of this interaction to be present throughout the cortex. These reconstructions (Fig. 5a,b) show pericyte processes extending from the cell body along the length of blood vessels and wrapping around them (Supplementary Video 5). We quantified three features in four vessels (Fig. 5c and Extended Data Fig. 8a). First, we looked at close pericyte–cEC interactions, in which the extracellular matrix was not visible between the pericyte and EC, finding a close interaction in ~83% of sections on average. Next, we analyzed ‘peg and socket’ interactions, membrane invaginations between ECs and pericytes. These interactions were rare in vessel 2 (13.8% of sections), appeared more frequently in vessels 1 and 4 (46% and 50% of sections, respectively) and were prominent in vessel 3 (85% of sections). Finally, we quantified pericyte contact with EC tight junction clefts, finding this interaction more frequently in vessels 1 and 2 (64% and 40% of sections) than in vessel 3 (25% of sections). In vessel 4, tight junction clefts were not detectable in the ~6.5 μm analyzed (Extended Data Fig. 8b). Together, these findings detail pericyte–cEC interactions along the length of a cortex capillary. Consistent with a previous report^39^, we found frequent pericyte interactions with EC tight junctions, while ‘peg and socket’ interactions were concentrated in smaller domains.

### Distinct molecular and structural features of ME pericytes

Although brain pericytes have generally been viewed as homogenous across brain regions in rodents and humans^40,41^, we identified several regional differences. First, we observed striking morphological differences by immunostaining and sparse labeling in *Pdgfrb*-CreERT2:Ai14 reporter mice (Fig. 5d,e, Extended Data Fig. 8c,d and Supplementary Video 6). Cortex capillary-associated pericytes showed a characteristic ‘bump on a log’ morphology (a prominent cell body with long, thin processes extending along vessels), whereas ME pericytes had a more irregular shape (a less defined cell body found between blood vessels with processes of varying lengths). Additionally, although we observed cortex pericytes frequently interacting along a single vessel, some ME pericyte processes contacted several blood vessels. This interaction is reminiscent of pericyte–EC interactions in peripheral organs like the pancreas^42^.

Several studies have shown that a lower ratio of pericytes to ECs is associated with higher brain blood vessel permeability^18,43,44^. To quantify pericytes and ECs, we performed pericyte-specific labeling. Although *Pdgfrb* labels pericytes, it can also label fibroblasts, which surround ME cECs. Fibroblasts also express *Pdgfra*. Thus, we performed immunostaining for EC nuclei (Ets transcription factor (ERG)) in *Pdgfra-*H2B-GFP; *Pdgfrb*-CreERT2:Ai14 tdTomato reporter mice, quantifying tdTomato^+^GFP^−^ pericytes and ERG^+^ ECs in each region. We found approximately half as many pericytes per EC in the ME than in the cortex (Fig. 5f), correlating with increased ME blood vessel permeability.

**Extended Data Fig. 9:**
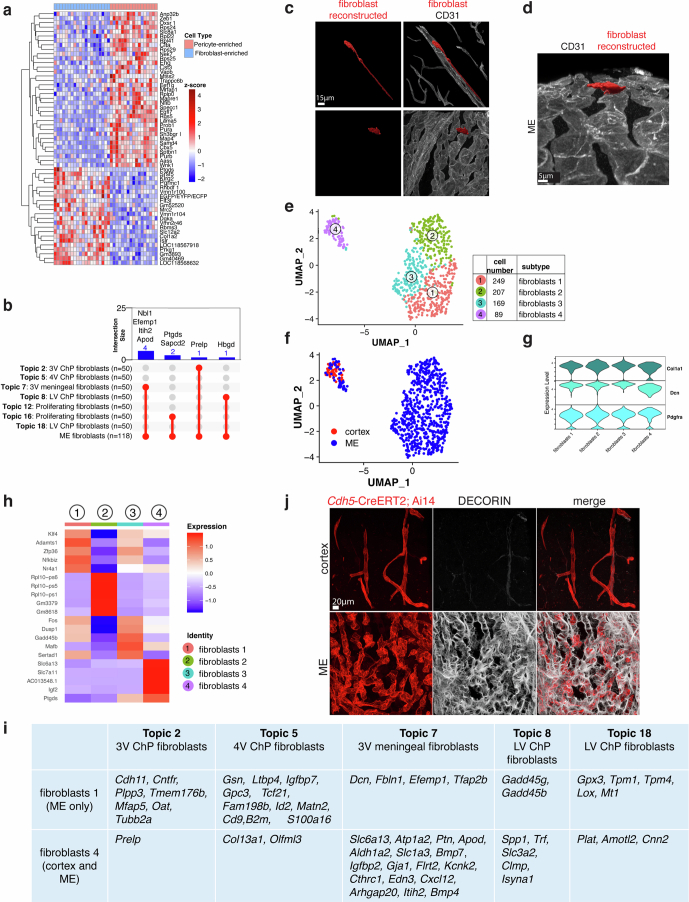
Fibroblasts in the ME. (**a**) Heatmap of differentially expressed genes between ME pericyte- and fibroblast-enriched areas of interest from GeoMX whole transcriptome profiling. (log_2_ fold-change greater than |1| and FDR < 0.05). Differential expression was determined by linear mixed model analysis and significance assessed by false discovery rate (FDR). (**b**) Upset plot showing overlap between ME fibroblast-enriched regions from GeoMx whole transcriptome profiling and fibroblasts from choroid plexus in Dani et al.^31^. (**c**) 3D reconstructions of fibroblasts in the ME and cortex are shown in red (based on *Pdgra-*CreER:Ai14 Tomato expression). Immunostaining for CD31 is shown in white. Scale bar 15 µm. (**d**) Another 3D view of (g) showing the location of fibroblasts below ME blood vessels. Scale bar 5 µm. (**e**) UMAP plot of 714 fibroblast transcriptomes. Fibroblast subtype clusters were identified with an unbiased analysis. The number of cells identified for each subtype is indicated in the plot legend. (**f**) UMAP plot in (a) colored by sample region. (**g**) VlnPlot showing the expression of *Cola1a*, *Dcn* and *Pdgfra* in fibroblast subclusters. (**h**) Heatmap illustrating the top 5 genes differentiating each fibroblast subcluster. Differentially expressed genes were determined by two-sided Wilcoxon test in Seurat comparing each subcluster to all other mural cell subclusters with min.pct=0.25 and log2_FC > 0.6 thresholds. (**i**) Table showing overlap of fibroblasts 1 and 4 subtypes with fibroblast topics from Dani et al.^31^. Differentially expressed genes were determined by two-sided Wilcoxon test in Seurat comparing the fibroblast 1 subcluster to the fibroblast 4 subcluster, with min.pct =0.25 and log2_FC > 0.6 thresholds. (**j**) Co-immunostaining for fibroblast-enriched protein, DECORIN (white) and Tomato in cortex and ME. Blood vessels labelled with Tomato using *Cdh5*-CreERT2:Ai14 mice. Scale bar 20 µm.

We next investigated molecular differences between ME and cortex pericytes using GeoMX whole transcriptome spatial profiling. As shown in Fig. 1a, leaky ME blood vessels are adjacent to non-leaky, BBB-containing blood vessels. Therefore, to gain higher resolution, we selected a spatial transcriptomic approach to unambiguously profile pericytes only from the ME region. As this method does not isolate single cells, we used *Pdgfra*-H2B-EGFP reporter mice and antibodies to distinguish fibroblasts (EGFP^+^), pericytes (DES^+^) and ECs (CD31^+^) (Fig. 5g). We selected pericyte-enriched areas around blood vessels in both regions and ME fibroblast-enriched areas as a control. We focused our analysis on genes expressed in pericytes (Methods and Supplementary Table 1). We confirmed pericyte enrichment based on expression of established markers (Extended Data Fig. 8e). We identified 137 differentially expressed genes between cortex and ME pericyte-enriched regions and 36 gene sets with differential enrichment by pathway analysis (Fig. 5h, Extended Data Fig. 8f and Supplementary Table 1). By immunostaining, we validated expression of one regionally enriched gene expressed in ME pericyte-enriched regions, *Slc12a7* (encoding KCC4) (Extended Data Fig. 8g). Finally, we compared genes expressed in ME pericyte-enriched samples to human lung^45^, gut^46^ and kidney^47^ mural cells and the recently described T- and M-pericytes in the human brain^40^. In total, 20 out of 65 ME differentially expressed genes (30.8%) were found in mural cells from another peripheral tissue compared to 13 out of 77 (16.9%) of cortex differentially expressed genes (Extended Data Fig. 8h). Differentially expressed genes between ME and cortex pericyte-enriched regions and pericyte marker genes from our scRNA-seq dataset did not show enrichment for markers of human T-pericytes and M-pericytes (Extended Data Fig. 8i,j), and ME pericyte-enriched and fibroblast-enriched regions showed gene expression differences (Extended Data Fig. 9a,b and Supplementary Table 1).

In short, pericytes show different morphologies and capillary coverage in the ME and cortex. Although brain pericytes show transcriptomic differences from pericytes in the periphery^19^, our analysis revealed that brain pericytes may also show transcriptomic differences across regions. Together, these findings suggest that like astrocytes, pericytes probably contribute to BBB functional differences between the cortex and ME through their interactions with cECs (elaborated in Fig. 7).

### Capillary-associated fibroblasts are present in the ME

**Fig. 6:**
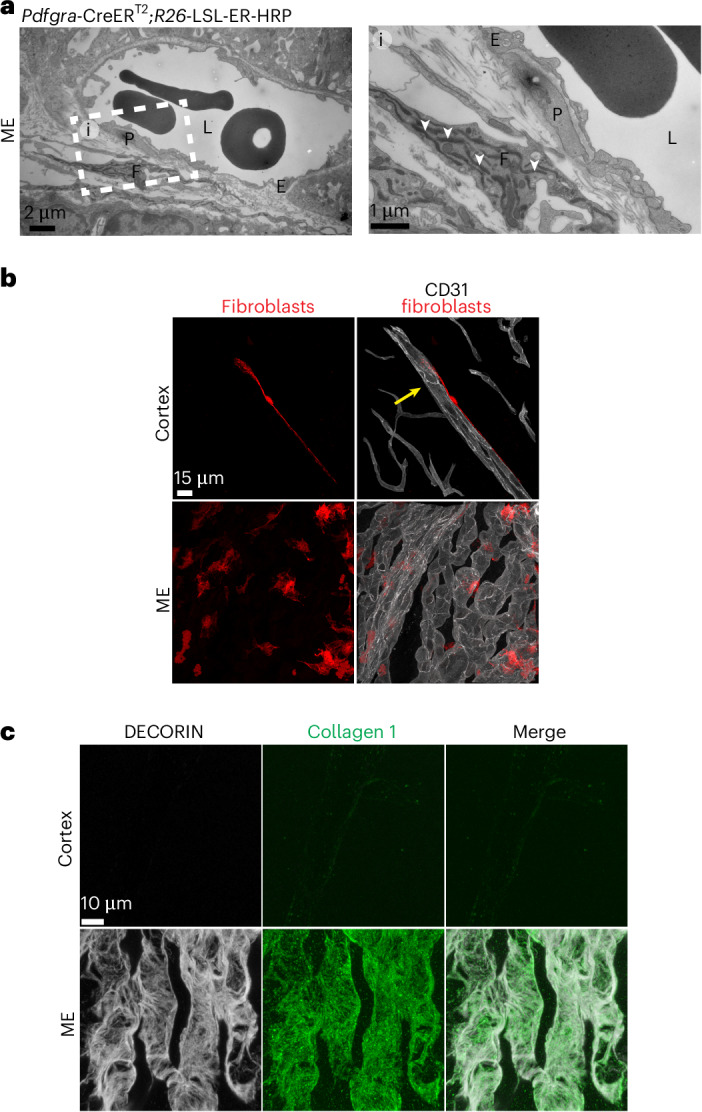
ME contains capillary-associated fibroblasts. **a**, TEM images of *Pdgfra*-CreER HRP reporter after DAB reaction in the ME. HRP is detected in the endoplasmic reticulum (white arrowheads) of *Pdgfra*-expressing fibroblast cells. **b**, Fluorescent labeling of fibroblasts in the cortex and ME using *Pdgfra*-CreER:Ai14 mice after low-dose 4OH-tamoxifen to achieve sparse cell labeling. Left: Tomato^+^ fibroblasts (red). Right column: merged with immunostaining for CD31 (white). Yellow arrow indicates artery. **c**, Co-immunostaining for fibroblasts with DECORIN (white) and collagen 1 (green) in cortex and ME.

Perivascular fibroblasts were observed previously in the cortex associated with large blood vessels^19^. Surprisingly, we found that only the ME contains numerous capillary-associated fibroblasts (Figs. 1g and 5f). To better characterize ME fibroblasts with TEM, we used a horseradish peroxidase (HRP) reporter driven by *Pdgfra-*CreERT2, finding fibroblasts in the ME extracellular space surrounding capillaries (Fig. 6a). We also used *Pdgfra*-CreERT2:Ai14 reporter mice to model fibroblast morphology with U.Clear. We found cortex fibroblasts along large vessels whereas ME fibroblasts were near capillaries, in accordance with our TEM data (Fig. 6b and Extended Data Fig. 9c,d).

Subclustering analysis of fibroblasts revealed three ME fibroblast subtypes (fibroblasts 1–3) and one subtype from the ME and cortex (fibroblast 4) (Extended Data Fig. 9e–h). Comparison of these subtypes to capillary-associated choroid plexus fibroblasts^31^ showed that ME-enriched subtypes exhibit similarity to third and fourth ventricle choroid plexus fibroblasts, whereas fibroblasts from subtype 4 are most similar to third ventricle meningeal fibroblasts (Extended Data Fig. 9i). Although all subtypes expressed *Pdgfra*, *Dcn* and *Col1a1*, we found that they were present only around ME capillaries (Fig. 6c and Extended Data Fig. 9j). Thus, the abundant fibroblasts near ME capillaries suggest that fibroblasts also have a role in regulating ME vascular permeability, perhaps by altering the composition of the extracellular matrix. Perivascular fibroblasts have also been observed near CNS capillaries in other CVOs and brain tumors^48–50^, indicating that fibroblasts may generally be associated with leaky CNS blood vessels in normal physiology and disease.

### Bioinformatic method finds candidate ligand–receptor pairs

**Fig. 7:**
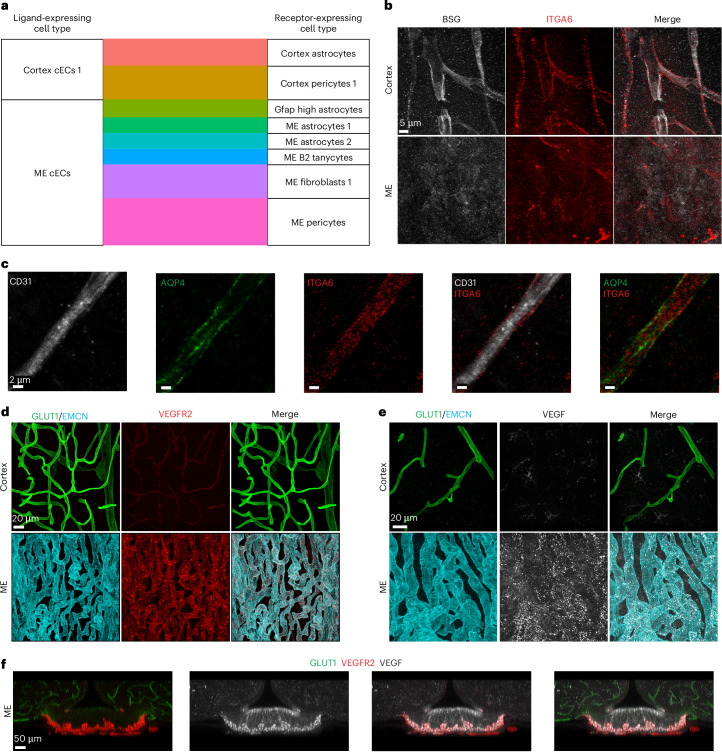
Differential intercellular signaling capacity identified in the ME and cortex. **a**, Alluvial plot showing the number of significant (*P* < 0.05) co-expressed EC ligands and perivascular receptors. **b**, Co-immunostaining for BSG (white) and its receptor integrin α6 (ITGA6, red) in cortex and ME, validating elevated expression of ligand (BSG) and receptor (ITGA6) in cortex. **c**, Co-immunostaining for CD31 (white), AQP4 (green) and ITGA6 (red) in cortex. **d**, Co-immunostaining for VEGFR2 (red), EMCN (cyan, ME ECs) and GLUT1 (green, cortex ECs) in cortex and ME. EMCN and GLUT1 were used to label ECs instead of CD31 owing to antibody compatibility with VEGFR2. **e**, Co-immunostaining for VEGF (white), EMCN (cyan, ME ECs) and (GLUT1, green, cortex ECs) in cortex and ME. EMCN and GLUT1 were used to label ECs instead of CD31 owing to antibody compatibility with VEGF. **f**, Immunostaining illustrating complementary spatial distribution of ligand VEGF (white) and receptor VEGFR2 (red) in ME. Non-ME vessels are labeled in green (GLUT1).

**Extended Data Fig. 10:**
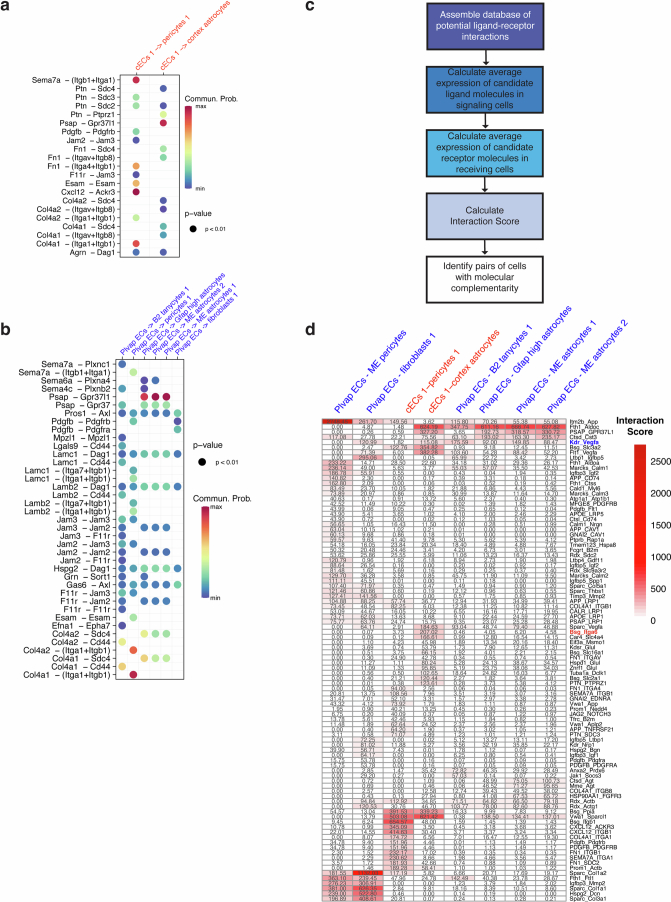
Differences in perivascular cell signaling capacity in the ME and cortex. (**a**, **b**) Bubble plots from CellChat analysis showing ligand-receptor pairs between (b) cortex ECs and pericytes and astrocytes and (c) ME Plvap ECs and astrocytes, tanycytes, pericytes and fibroblasts. ME pericyte data is from the scRNAseq dataset. p-values were calculated by permutation test in the CellChat R package. (**c**) Overview of ligand-receptor analysis methodology and workflow. (**d**) EC ligand-receptor interaction scores with pericyte, astrocyte, tanycyte and fibroblast receptors in the ME or cortex with values > 40 and p-values < 0.01. Candidate, ME-enriched ligand-receptor interaction investigated in Fig. 7 is highlighted in blue. Candidate, cortex-enriched ligand-receptor interaction investigated in Fig. 7 is highlighted in red. Uppercase ligand-receptor pairs are from Kumar et al. database. ME pericyte data is from GeoMX spatial transcriptomic profiling.

The proximity and direct physical interactions between cECs and perivascular cells suggested the feasibility of ligand–receptor interactions between these cells as a mechanism to regulate local blood vessel permeability. Indeed, a recent study showed such an interaction between pericytes and cECs at the BBB. Specifically, pericyte-secreted vitronectin interacts with receptor integrin alpha 5, expressed in neighboring cECs, to actively suppress transcytosis in cECs and thus maintain BBB integrity^51^. Similarly, recent studies indicate that the CVO environment contains cues to actively regulate leakiness^22,23^. To unbiasedly identify ligand–receptor pairs that may support intercellular signaling to regulate blood vessel permeability, we used CellChat^52^ and a co-expression method^53^. For the co-expression method, we sought to identify new interactions, supplementing a published database of experimentally validated interactions with predicted interactions of differentially expressed genes (Methods). By both methods, we identified known interactions important for the BBB, like PDGFβ–PDGFRβ between cECs and pericytes. Using CellChat, we found 25 and 33 enriched ligand–receptor pairs between cECs and pericytes in the cortex and ME, respectively, and 35 and 20 enriched ligand–receptor pairs between cECs and astrocytes in the cortex and ME, respectively (Extended Data Fig. 10a,b and Supplementary Table 1). Using the co-expression method, we found 37 and 62 enriched ligand–receptor pairs between cECs and pericytes in the cortex and ME, respectively, and 21 and 17 enriched ligand–receptor pairs between ECs and astrocytes in the cortex and ME (ME 1 astrocytes), respectively (Fig. 7a, Extended Data Fig. 10c,d and Supplementary Table 1). We confirmed the co-expression of one candidate cortex EC–astrocyte ligand–receptor pair: *Bsg* and *Itga6*. Immunostaining shows BSG expression in cortex cECs but not in ME cECs, and robust ITGA6 expression in cortex astrocyte endfeet—in addition to ECs—but decreased perivascular expression in the ME (Fig. 7b,c). Finally, extending our analysis to other ME cell types, we confirmed the expression of VEGFR2 (encoded by *Kdr*) in ME cECs and found co-expression of its ligand (VEGFA) in ME-specific tanycyte cells (Fig. 7d–f), in line with both methods.

This in silico analysis evaluated ligand–receptor expression patterns to identify candidate pericyte-derived and astrocyte-derived factors that may act upon ECs to maintain BBB integrity. Moreover, the co-expression method provides a molecular handle for future strategic investigation of EC–perivascular cell interactions. Future experimental characterization of this intercellular signaling, together with the fast-growing identification of additional signaling pathways in cECs, will provide a comprehensive understanding of local BBB regulation and heterogeneity.

## Discussion

We combined scRNA-seq, spatial transcriptomic profiling, TEM and serial EM reconstruction, and U.Clear imaging to compare the vascular microenvironment of two brain regions showing BBB heterogeneity. In addition to molecular and morphological specializations of ECs and perivascular cells, we found distinct cell organization and identified putative ligand–receptor pairs that may mediate cell–cell signaling in the ME and cortex. These findings support the idea that intracellular signaling within ECs and intercellular signaling between ECs and perivascular cells control brain cEC permeability. This is in line with previous work showing that molecules like MFSD2A regulate permeability within ECs^8,54^, and that intercellular signaling with astrocytes through the Wnt pathway^55^ and pericytes through vitronectin–integrin regulate BBB formation^51^. This work serves as a foundation, revealing how alterations in cell interactions may control local blood vessel permeability and demonstrating the importance of performing both molecular and morphological characterizations to understand BBB properties.

The CVOs are key sites for body–brain communication. The leaky nature of CVO blood vessels permits rapid bidirectional communication between the circulation and the brain. Neurons that sense signals through the CVOs are being found to perform increasingly important and diverse functions related to body physiology regulation in health and disease. Multiple CVOs communicate with the hypothalamus^56,57^, a central regulator of temperature and sickness behavior during infection^58,59^, to coordinate humoral responses to environmental stimuli by affecting feeding behaviors, cardiovascular function and body temperature. Recent work also shows that CVOs may serve as an immune entry site in disease models^60^. This CVO vascular atlas can provide information to help understand how body–brain communication is achieved in this area to perform these essential functions. It has been also observed that blood vessels adjacent to the ME show increased leakiness during the physiological response to hunger^3,61^. Our molecular profiling of ME perivascular cells may facilitate future investigation of such plasticity by uncovering molecules and cell types involved in regulating CVO permeability.

Our transcriptomic analyses also lend further support to the idea that increased vascular permeability in the CNS is actively regulated and maintained by extrinsic factors in the local environment. Blood vessels in other CVOs, the choroid plexus and the choroid capillaries of the eye also show increased permeability^2,62^. Recent studies indicate that the permeability of these specialized vessels is not caused by a lack of barrier induction but is actively induced by the local microenvironment^63^. In choroid capillaries, VEGF secreted by the retinal pigment epithelium^64^ and inhibition of Wnt signaling regulate blood vessel permeability^23^. In the zebrafish pituitary, blood vessel permeability is induced by *Cyp26b1*, *Tgfb* and VEGF derived from pituicytes^65^. In the mouse area postrema, *Wif1*, which blocks Wnt activation, is expressed^23^. Although in all cases, permeability is locally induced in the perivascular environment, the cell source of these signals differs. By systematically analyzing ME cell–cell interactions, we found that VEGF is expressed in ME astrocytes and tanycytes and that ME cECs have enriched expression of VEGFR2. Additionally, in accordance with suppressed Wnt signaling in ME cECs (Extended Data Fig. 5f). The Wnt inhibitor *Sfrp5*^66^ was expressed in ME astrocytes (Extended Data Fig. 7g). Moreover, the presence of ME capillary-associated fibroblasts, which are found in other leaky CNS regions, suggests that the extracellular matrix may also have a role in promoting vascular permeability in these regions.

Finally, this work revealed how performing scRNA-seq on small regions can uncover information about cell heterogeneity and the specialization of rare cell types within the brain. This platform can be applied in other brain regions to clarify how regional differences in cell organization and signaling affect BBB properties. Most CNS diseases affect specific brain regions. Therefore, alterations in cell signaling in disease could be driven by differences in the local composition or interactions of perivascular cells. Identifying the factors underlying BBB heterogeneity is an important step toward developing targeted therapies to make disease treatment as region-specific as possible.

## Methods

### Mice

All mouse experiments followed institutional and US National Institutes of Health guidelines and were approved by the Harvard University Institutional Animal Care and Use Committee. Mice were maintained on a 12 h light:12 h dark cycle at 22 °C and 55% humidity. Mice used in all experiments were 8–14 weeks old; both male and female mice were used unless otherwise indicated. The following mouse strains were used: wild type (C57BL/6N, Charles River Laboratories, strain 027), Ai14 (JAX, strain 007914)^67^, *Aldh1l1*-EGFP (JAX, strain 026033)^68^, GFAP-GFP (JAX, strain 003257)^37^, *Glast*-CreER (JAX, strain 012586)^69^, TCF/LEF-GFP (JAX, strain 032577)^28^, *Cdh5*-CreERT2^70^, *Slco1c1*-CreERT2^33^, *Pdgfrb*-CreERT2 (JAX, strain 029684)^71^, *Pdgfra-*H2B-EGFP (JAX, strain 007669)^72^, *ROSA26*^*LSL-ER-HRP*^ (JAX, strain 034746) and *Mfsd2a*^ko^ (MMRRC, strain 032467-UCD)^73^.

Heterozygous *Glast*-CreER, *Pdgfrb*-CreERT2, *Slco1c1*-CreERT2 or *Cdh5*-CreERT2 mice were crossed with homozygous Ai14 mice to generate CreER-dependent reporter mice. Cell-type labeling was induced by five subsequent intraperitoneal injections of tamoxifen (1 mg per mouse). Brains were collected 2 weeks later. For sparse cell labeling, a single dose of 4OH-tamoxifen (0.4 mg per mouse) was injected 1 week before analysis. Given that the GLAST reporter can occasionally label tanycytes, which express *Slc1a3* at a lower level than astrocytes, we distinguished tanycytes from ME astrocytes by morphology, expression of vimentin and cell body location relative to the third ventricle (Fig. 4d, Extended Data Fig. 7k,n,o and Supplementary Video 4).

### U.Clear tissue clearing

U.Clear tissue clearing is a newly optimized protocol based on the Adipo-Clear framework^74,75^. In brief, mice were deeply anesthetized with ketamine and xylazine (100 mg kg^−1^) and subsequently intracardially perfused with cold 4% paraformaldehyde (PFA) in PBS. Brains were dissected and fixed overnight in 4% PFA at 4 °C. For claudin-5 staining, brains were perfused with cold PBS and drop-fixed in cold 100% methanol overnight before rehydrating brains in a series of 70% methanol–PBS, 30% methanol–PBS and PBS. After PBS washing, brains were dissected into a 5 × 5 × 5 mm cube of somatosensory cortex and a similarly sized cube of hypothalamus containing the ME. The resulting samples were delipidized by four washes (1 h, 2 h, 4 h, overnight) with SBiP buffer (200 µM Na_2_HPO_4_, 0.08% sodium dodecyl sulfate, 16% 2-methyl-2-butanol, 8% 2-propanol in H_2_O (pH 7.4)) at room temperature (~22 °C). Next, samples were transferred into B1n buffer (0.1% Triton X-100, 2% glycine, 0.01% 10 N sodium hydroxide, 0.008% sodium azide in H_2_O) for blocking under nutation at room temperature. On the next day, samples in B1n buffer were incubated at 37 °C for 1 h. For immunolabeling, samples were incubated at 37 °C for 2 days in primary antibodies diluted in PTxwH buffer (0.1% Triton X-100, 0.05% Tween-20, 0.002% heparin (w/v), 0.02% sodium azide in PBS) with gentle rocking. Samples were then washed four times with PTxwH (1 h, 2 h, 4 h, overnight). Samples were then incubated at 37 °C for 2 days in secondary antibodies diluted in PTxwH with gentle rocking and subsequently washed four times with PTxwH (1 h, 2 h, 4 h, overnight). For further delipidization, samples were immersed in SBiP buffer four times (1 h, 2 h, 4 h, overnight). Next, samples were washed twice in 0.5 mM Na_2_HPO_4_ (1 h, 2 h), twice in PB buffer (16 mM Na_2_HPO_4_, 4 mM NaH_2_PO_4_ in H_2_O) (1 h, 2 h) and finally twice in PTS solution (75% PB buffer, 25% 2,2’-thiodiethanol) (1 h, overnight), then equilibrated with histodenz gradient buffer with refractive index adjusted to 1.53 using thiodiethanol. Samples were stored at −20 °C until acquisition. To validate that BBB organization and morphology were intact following U.Clear, we performed a comparison to thick tissue sections (Extended Data Fig. 1f).

### Immunohistochemistry

For anti-KCC4 (*Slc12a7*) and anti-CD31 co-immunostaining, wild-type brain tissue was perfused and fixed in 4% PFA and PBS as described above. The tissue was washed three times in PBS, equilibrated in 30% sucrose and PBS at 4 °C and flash-frozen in NEG-50 for cryosectioning. Then, 25 μm coronal sections were blocked for 1 h at room temperature in PBS plus 10% normal donkey serum and 0.1% Triton X-100, then incubated in blocking buffer with primary antibodies overnight at 4 °C. Samples were washed three times in PBS with 0.1% Triton X-100, then incubated in blocking buffer with secondary antibodies for 1 h at room temperature. Samples were washed three times in PBS with 0.1% Triton X-100 and stained with DAPI.

### Antibodies

Primary and secondary antibodies used in this study are detailed in Supplementary Table 2.

A polyclonal antibody to the carboxyl terminus of mouse Mfsd2a was generated by New England Peptide using Institutional Animal Care and Use Committee-approved protocols. Rabbits were immunized with a KLH-conjugated peptide (Ac-CSDTDSTELASIL-OH). Antiserum was purified by peptide affinity column. Antibody specificity was validated in *Mfsd2a*^ko^ mice (Extended Data Fig. 1e).

### Light microscopy

Cleared and stained brains and tissue sections were analyzed at high resolution with a Leica TCS SP8 confocal microscope. U.Clear *Z*-stacks were processed and 3D-reconstructed with Imaris software (v.9.3.1; Oxford Instruments). Immunostained sections were processed with FIJI (v.2.1.0). Photoshop CC and Illustrator CC (Adobe) were used for image formatting.

### Image analysis

Capillary diameter, EC density and EC pericyte coverage were quantified from three ~50 µm-thick 40× confocal stacks of capillaries in cortex and ME per mouse. To measure capillary thickness, the area covered by three different capillaries in each image, labeled by CD31 immunostaining, was measured and divided by their respective vessel length. The average of these three diameters was used as the average capillary length for an image, represented as a single data point on a graph. To measure EC nuclei per vessel length, ERG^+^ EC nuclei were counted and total capillary length was measured. All analysis was performed blinded. Each data point in the graph represents an individual image. To measure pericyte coverage, EC nuclei were labeled with ERG antibody. Pericytes (GFP^−^, Tomato^+^) were identified using *Pdgfra*-H2B-GFP; *Pdgfrb*-CreERT2:Ai14 mice. All analysis was performed blinded. Each data point in the graph represents an individual image. In all analyses, data points from the same mice are depicted in the same color, values are shown as mean ± s.d. and significance was determined using a nested two-tailed *t*-test in GraphPad Prism (v.8). Data distribution was assumed to be normal, but this was not formally tested.

### Tracer permeability assays

EZ-Link sulfo-NHS-LC-biotin tracer was injected retro-orbitally under short isoflurane anesthesia (0.2 mg g^−1^ bodyweight). Brains were dissected 30 min after injection after perfusion with 4% PFA as described above.

### scRNA-seq and analysis

#### Sample isolation and dissociation

For each experimental replicate, cortex and ME were isolated from five 9-week-old male mice, pooled and processed together. Male mice were used to limit variations in the ME by the estrous cycle, as the ME is responsive to reproductive hormones^2^. Mice were killed at 08:00 h to avoid circadian cycle variation. Dissociation into single cells was performed using a protocol adapted from a previous publication^76^. Brains were dissected in ice-cold dissociation medium (DM; 1× Hank's balanced salt solution without calcium and magnesium, 0.01 M HEPES, 9 mM MgCl_2_, 35 mM d-glucose pH 7.35). First, the ME was removed then the brain was cut into 1 µm thick sections by sectioning matrix (Ted Pella). Cortex samples were obtained with a 1 mm biopsy puncher (Harris Uni-Core). Samples were dissociated using the papain dissociation system (Worthington) according to the manufacturer’s instructions with the following modifications. Dissociation was performed at 37 °C for 45 min with gentle agitation in DM plus papain (20 U ml^−1^) and DNase (200 U ml^−1^). After centrifugation with ovomucoid inhibitor in DM, cells were washed in DM containing 0.04% BSA and resuspended in DM with 0.04% BSA and 15% Optiprep (Sigma-Aldrich) for inDrops cell encapsulation, performed by the Single Cell Core at Harvard Medical School.

#### inDrops library preparation, sequencing and data processing

Two replicates of approximately 3,000 cells were collected from each experimental sample. inDrops was performed as described previously^25,26^ using v3 barcodes, generating 22 and 30 libraries from cortex and ME samples, respectively. Libraries were pooled and sequenced in 21 runs with the NextSeq 500 using the high output flow cell (Illumina), pooling 3,000 to 12,000 cells per sequencing run. Transcripts were processed with bcbio-nextgen inDrops3 data pipeline (v.1.2.8) using the default parameters.

#### Quality control and filtering

Analysis was performed with R^77^ (v.4.0.2) in RStudio^78^ using the Seurat analysis package (v.4.0.2)^79–81^. In each library, empty droplets were predicted using the EmptyDrops function^82^ and doublets were predicted with scrublet^83^. The levels of ambient RNA in ME and cortex were estimated separately using SoupX^84^. In brief, before filtering, all samples from each region were merged into a single dataset. Each dataset was clustered using the default Seurat analysis parameters to assign tentative cluster identities. Using the assumption that the background profile is the same as the average expression across all cells, we performed a custom estimation of the soup profile using the ‘setSoupProfile’ command. The corrected count matrices were then merged to generate the combined dataset.

To select for high-quality cells, we filtered based on number of genes expressed (at least 500), number of reads per cell (at least 750), percentage of mitochondrial genes (≤15%) and, by novelty index, the ratio of the number of genes detected to the number of reads for each cell (≥0.4). Clustering analysis was performed using the default Seurat analysis pipeline. In brief, the dataset was log-normalized and scaled to 10,000 transcripts per cell. Highly variable genes were determined with the Vst selection method using the default 2,000 features. All genes were then scaled across all cells so the mean expression of each gene is 0 and the variance of each gene is 1. Next, principal component analysis was performed using the calculated variable features.

The top 30 principal components were used in downstream steps based on the output of the ElbowPlot function. Clustering was performed at resolution 0.5 to identify broad cell types, resulting in 35 clusters. After clustering, cells predicted to be empty droplets (EmptyDrops output false discovery rate (FDR) > 0.01) and doublets (scrublet output score of >0.25) were removed from the dataset. Additionally, one cluster in which more than 70% of the cells had a doublet score of >0.25 and showed expression of marker genes of both neurons and oligodendrocytes was removed from the dataset. The dataset was then re-analyzed as above without these cells, resulting in 104,091 cells and 33 clusters. After subclustering analyses (described below), the dataset was re-analyzed to generate the final dataset of 58,117 cells in 30 clusters with an average number of unique molecular identifiers per cell of 4,283 and an average number of genes per cell of 2,197. We defined clusters as regionally enriched by the scProportionTest algorithm^85^ (v.0.0.0.9000).

#### Cell type assignment and subclustering analyses

Marker genes for each cluster were determined with the FindAllMarkers function using the default Wilcoxon test and the following parameters: only.pos = TRUE, min.pct = 0.25, logfc.threshold = 0.25. To assign cell types, known marker genes were used (Supplementary Table 3).

#### Classification of cell subtypes

For iterative subclustering, analysis was performed as for the complete dataset described above unless indicated. Clusters that expressed marker genes characteristic of multiple cell types or that were isolated from only one sample were removed (described below), and the data were reclustered until all subtype clusters showed expression of known marker genes for the given cell type. ECs, astrocytes, neurons, oligodendrocytes, mural cells, fibroblasts and pars tuberalis cells were subclustered individually; tanycytes and ependymal cells, and microglia, PVMs and T cells were subclustered together.

##### ECs

ECs were subclustered four times to remove contamination from SMCs and pericytes and to remove clusters with ribosomal marker genes. This analysis resulted in seven subclusters that correspond to cECs, vECs, aECs, *Plvap*^+^ ECs and tip cells (Fig. 3 and Extended Data Fig. 4). aECs express the marker genes *Bmx* and *Vegfc*; vECs express marker genes *Lcn2* and *Nr2f2*; and cECs lacked expression of arteriolar and venous marker genes and expressed marker genes *Mfsd2a* and *Tfrc*. cECs 1 and 2 differ in their expression of immediate early genes, probably because of EC activity-induced transcription^76^ or activation following tissue dissociation^86^. aECs 1 express arteriolar markers at a lower level than aECs 2, suggesting that they represent ECs at the capillary-to-arteriolar transition.

##### Mural cells

To identify mural cell subtypes, mural cells were subclustered three times (first at resolution 0.5, then at resolution 2) to remove clusters with EC marker genes, resulting in four clusters that include two pericyte subclusters and two smooth muscle cell clusters. One cluster was removed that expressed both EC and pericyte marker genes, considered doublets. The remaining clusters express pericyte or SMC marker genes, with or without activation markers.

##### Astrocytes

Subclustering analysis was performed first at resolution 2, then at resolution 0.4 to remove clusters expressing neuron and oligodendrocyte marker genes and ribosomal marker genes, resulting in four subclusters.

##### Fibroblasts

Initial subclustering analysis of fibroblasts (resolution 2, 20 principal components) revealed 12 subclusters. Subclusters expressing EC marker genes were removed and the dataset was reclustered (resolution 0.5, 20 principal components), resulting in four subclusters. The smallest subcluster contained cells from only one experimental replicate. These cells were removed and the data were reclustered (resolution 0.5, 20 principal components) to reveal four subclusters.

##### Microglia, PVMs and T cells

Subclustering analysis removed subclusters with vEC, oligodendrocyte and astrocyte marker genes, resulting in eight immune cell subtypes^87,88^.

##### Tanycytes and ependymal cells

Subclustering of tanycyte and ependymal cells (20 principal components, resolution 0.5) removed clusters with oligodendrocyte or PVM marker genes or ribosomal marker genes, revealing eight ME-derived subclusters, with subtypes consistent with previous reports^89^.

##### Oligodendrocytes

Subclustering analysis of oligodendrocytes removed astrocyte and immune cell contamination, revealing 12 subclusters, consistent with previous reports^36,90^.

##### Neurons

Subclustering analysis removed astrocyte and oligodendrocyte contamination and low-quality clusters with *Malat1* or ribosomal genes as marker genes, resulting in 23 subclusters; 10 from the cortex and 13 from the ME.

##### Pars tuberalis

Subclustering analysis removed clusters with neuron, astrocyte and keratinocyte contamination, resulting in four subclusters.

#### Pathway enrichment analysis

Pathway enrichment analysis was performed as previously described^91^. In brief, curated gene sets (C2) and cell type signature gene sets (C8) were downloaded from the MSigdB (v.7.5.1)^92,93^; mouse gene IDs were converted to human homologs using SynGO^94^. Differentially expressed genes were calculated in Seurat using the two-sided Wilcoxon test as indicated. Pathway enrichment was determined using bc3net^95^ (v.1.0.4) with default parameters and plotted with the pheatmap function (v.1.0.12) as −log of the adjusted FDR.

#### Differential gene expression analysis and comparison to pituitary gland, neurohypophysis and peripheral ECs

Differentially expressed genes between cortex and ME cECs were calculated with the FindMarkers function (Wilcoxon test, min.pct > 0.25, avg_log_2_FC > 0.6). The intersection of the top 100 enriched genes in ME cECs, the top 100 marker genes in vascular ECs from the mouse neurohypophysis^32^ and the top 100 genes in pituitary ECs^23^ was displayed using ggvenn (v.0.1.9). For ECs from peripheral tissues, the top 50 enriched genes were compared to the top 50 marker genes from ME cECs, tip cells and cortex-derived cECs 1 subtypes. Barplots show the per cent overlap of the top 50 genes. Finally, the overlap between all enriched genes in kidney cECs, pancreas cECs^30^ and choroid plexus ECs^31^, and ME cECs-enriched differentially expressed genes was displayed using ggvenn (v.0.1.10).

#### Integration analysis of astrocytes and comparison to astrocyte subtypes

Integration analysis was performed with Harmony^34^ in Seurat using the RunHarmony function. Genes differentially enriched in astrocyte subtypes in a previous publication^35^ were visualized with VlnPlot and FeaturePlot features in Seurat. For comparison to aggregate metacell astrocyte subtypes from another publication^36^, astrocyte subtype aggregated expression was calculated with the AggregateExpression function in Seurat. Differentially expressed genes in astrocyte subtypes from this study were determined with the FindAllMarkers function (Wilcoxon test, min.pct > 0.25, avg_log_2_FC > 0.6), and samples from both studies were clustered based on the expression of these genes with pheatmap. Expression of the top 15 genes in the most similar astrocyte subtypes in a previous publication^36^ was also visualized with pheatmap.

### GeoMX spatial whole transcriptomic profiling

#### Tissue preparation

*Pdgfra*-H2B-EGFP mice were anesthetized with ketamine and xylazine, then transcardially perfused with 15 ml of ice-cold PBS followed by 50 ml of ice-cold fixative solution (4% PFA in PBS). Brains were extracted and post-fixed for 3 × 20 min, then 4 h in fixative solution on ice. Brains were further fixed in fixative solution at 4 °C overnight, then for a final 4 h the next day. Brains were washed three times for 5 min in PBS before paraffin embedding. Then, 5 μm sections containing the ME and/or cortex were mounted on SuperFrost slides (Fisherbrand).

#### GeoMX digital spatial profiling

For digital spatial profiling (DSP), spatial transcriptomics was performed in the NanoString GeoMx DSP platform using the mouse whole transcriptome atlas for >19,000 transcripts of protein-encoding genes. The DSP workflow was carried out by NanoString Technologies through the Technology Access Program.

Slides were baked, deparaffinized and rehydrated using graded ethanol. Target epitope retrieval was performed with Tris-EDTA (pH 9.0), then proteinase K to expose RNA targets. In situ hybridization with the whole transcriptome probes was performed overnight. The next day, slides were washed to remove off-target probes and samples were stained with morphology markers, to distinguish ECs, pericytes and fibroblasts (Supplementary Table 2), and Syto83 (1:10; Invitrogen) to visualize cell nuclei. Fluorescent images were collected by a GeoMx DSP instrument for region of interest selection.

ME and cortex DES^+^GFP^−^ areas (pericyte segments) and ME DES^−^GFP^+^ areas (fibroblast segments) were collected for transcriptional profiling. Labeled cells associated with large blood vessels were excluded as much as possible to reduce the collection of vascular smooth muscle cells. Additionally, samples were selected from the middle of the ME region to avoid smooth muscle cells (Extended Data Fig. 5a–c). In total, samples from eight animals of both sexes were profiled over three separate days for a total of 79 segments. Ultraviolet light was used to photocleave antibodies and release oligodendrocytes from areas of interest. Oligodendrocytes were collected and quantified by next-generation sequencing, and reads were processed into digital counts for data analysis.

#### DSP analysis

Data were analyzed in R (v.4.1.2) using GeomxTools (v.3.1.1).

##### Quality control

First, all segments passed a sequencing quality control assessment. Next, negative control probes were used to estimate background and downstream gene detection and to remove outliers. The limit of quantification of negative control probes in each region of interest was calculated to identify genes detected above background.

Several segments were removed because of low gene detection (<10% of the probes detected). Samples from six animals remained for analysis. Gene filtering was performed, resulting in 7,844 targets detected above the limit of quantification in 30% or more segments. Owing to differential distribution of cell types in the ME and cortex (Supplementary Table 1), genes attributed to astrocyte, L5.PT.CTX and EC profiles from a published study^96^ and genes from ME cECs, cortex cECs and tip cell subtypes were removed from the analysis (unless they overlapped with the annotated pericyte profile). A total of 2,921 genes remained from 53 of the 79 segments from four animals. Quartile three normalization was performed for genes in each segment. Principal component analysis followed by dimensional reduction showed that ME segments cluster separately from cortex segments and that ME pericytes and fibroblasts cluster separately. Coefficient of variation analysis of the top 292 genes (90^th^ quantile) showed that these genes cluster by region and cell type.

##### Differential gene expression and pathway analysis

Differential gene expression analysis was performed on a per-gene basis, modeling normalized gene expression using a linear mixed-effect model to account for the sampling of multiple segments from each tissue. To compare ME and cortex pericyte segments, the following formula was used: gene ~ pericyte region segment + (1 + pericyte region segment per animal). To compare ME pericyte and fibroblast segments, the following formula was used: gene ~ cell type segment + (1 + cell type segment per animal). Differentially expressed genes were considered significant at a FDR < 0.05 and |log_2_(fold change)| > 1. Differentially expressed genes were compared to enriched genes from human mural cells from the lung^45^, gut^46^ kidney^47^ and brain^40^ and visualized by UpSetR (v.1.4.0)^97^. Differentially expressed genes were also compared to mouse choroid plexus pericytes^31^ and lung pericyte-enriched genes reported previously^19^. Finally, pathway analysis was performed with GSVA (v.1.46.0)^98^ using the KEGG BRITE database. A total of 337 gene sets were scored, with each gene set containing 5 and 500 genes. Enriched pathways were significant at FDR < 0.05. Plots were generated with the EnhancedVolcano (v.1.6.0) and UpsetR packages. We also found enrichment of eight gene sets and 22 genes in ME fibroblasts relative to ME pericytes (Extended Data Fig. 9a,b and Supplementary Table 1), including *EGFP* and cortex fibroblast markers *Islr* and *Ptgds*.

### TEM

Mice were anesthetized with ketamine and xylazine, then transcardially perfused with cold, 5% glutaraldehyde, 4% PFA and 0.1 M sodium cacodylate. Brains were dissected and post-fixed overnight at 4 °C in fixative solution. Brains were then washed overnight in 0.1 M sodium cacodylate. Brains were washed three times for 15 min in 0.1 M sodium cacodylate, then sectioned into 100 μm coronal sections by vibratome. HRP reporter strains were processed with diaminobenzidine as described previously^99^. Regions of interest were microdissected, post-fixed in 1% osmium tetroxide and 1.5% potassium ferrocyanide, dehydrated and embedded in epoxy resin. Ultrathin sections of 80 nm were cut from the block surface, collected on copper grids and counter-stained with Reynold’s lead citrate before examination under a 1200EX electron microscope (JEOL) equipped with a 2k CCD digital camera (AMT).

### Serial TEM reconstruction

Serial TEM data of the visual cortex was generated previously (450 μm × 450 μm × 50 μm volume)^38^. Capillaries were reconstructed in FIJI^100^ using TrakEM2 (ref. ^101^). ECs and pericytes were manually traced in each section in the dataset, then the images were rendered together to create a 3D reconstruction. Vessel 1 was reconstructed through 794 serial 40 nm sections or 31.8 μm, with 66 sections excluded. Vessel 2 was reconstructed in 490 serial 40 nm sections or 19.6 μm, with 28 sections excluded. Renderings were processed in blender (blender.org). Two additional vessels were analyzed for interaction features: vessel 3 over 22 μm and vessel 4 over 6.5 μm.

### Ligand–receptor analysis

We analyzed our dataset with CellChat^52^ (v.1.6.0) and performed an analysis similar to that in a previous publication^53^. In brief, a ligand–receptor database was assembled. To facilitate the discovery of novel ligand–receptor interactions, the reference database was supplemented with differentially expressed genes in cECs and astrocytes (Supplementary Table 1). The predicted subcellular localization of each gene was determined using the Uniprot database^102^. For those genes known or predicted to be localized to the plasma membrane, secreted proteins or extracellular matrix components, the STRING database^103^ was queried to identify candidate interaction partners. Interaction partners with experimental validation were added to our ligand–receptor database. Established interactions^53^ are displayed in uppercase (for example, ‘PDGFB–PDGFRB’) and candidate ligand–receptor pairs are displayed in lowercase (for example, ‘Bsg–Itga6’). An interaction score was calculated for each ligand–receptor pair for two candidate interacting cell subtypes of interest by multiplying the average expression of the ligand gene in the candidate signaling cell and the average expression of the receptor gene in the candidate receiving cell. ME pericyte average expression data was generated by coercing the GeoMX data into a Seurat object (R v.4.1.3). An interaction score cutoff was determined by bootstrapping. In brief, the average expression of all genes in each cell subtype was calculated. For each iteration, the dataset was randomized with replacement, and interaction scores were calculated between the ligand-expressing cell subtype of interest and 2,192 random genes (the size of the supplemented database). This iteration was repeated 1,000 times to generate a null distribution of interaction scores. We focused on interaction scores >40, as these values were observed with a one-sided *P* value of <0.01 after Bonferroni correction for multiple comparisons (Supplementary Table 1). For the determination of unique ligand–receptor pairs in pericytes, pairs from the ME that were above this threshold in all cell types except ME pericytes were excluded, as we expect that they are probably a result of methodological differences.

### Statistics and reproducibility

Data collection and analysis were performed blind to the conditions of the experiments where indicated. All representative immunofluorescence and TEM images were performed in three or more mice and repeated in at least three independent experiments. For confocal and electron microscopy data, we performed preliminary experiments to identify the variation. We then performed a power test to identify appropriate sample sizes of images per mouse.

A total of 52 total inDrops scRNA-seq samples were collected on 15 separate days, with two technical replicates from ME and cortex samples on each day (except for days 12–15, which were ME only). Sequencing libraries were generated over 9 days to minimize variation owing to library preparation. For GeoMX DSP, samples from eight animals of both sexes were profiled over three separate days. For transcriptomic experiments, sample sizes were chosen based on the yield of high-quality vascular cells. For scRNA-seq, we aimed to profile at least 100 cells per cluster from each region of our cell types of interest. For GeoMX, we based our sample size on the reproducible clustering of samples from multiple animals on separate experiment days both by sample region and enriched cell type.

### Reporting summary

Further information on research design is available in the Nature Portfolio Reporting Summary linked to this article.

## Online content

Any methods, additional references, Nature Portfolio reporting summaries, source data, extended data, supplementary information, acknowledgements, peer review information; details of author contributions and competing interests; and statements of data and code availability are available at 10.1038/s41593-024-01743-y.

## Supplementary information

Supplementary Information Supplementary Tables 1–3, Supplementary Videos 1–6, Supplementary Figs. 1–3

Reporting Summary

Supplementary Table 1 scRNAseq cell type- and subtype-enriched genes, pathway analysis, spatial transcriptomics data and ligand–receptor interaction scores

Supplementary Video 1 **Sulfo-NHS-biotin leakage in cortex and ME**. Immunostaining for EC marker CD31 (white) and BBB leakage tracer (sulfo-NHS-biotin, magenta) showing no leakage of tracer in cortex and tracer leaking out of vessels in ME.

Supplementary Video 2 **Morphology of capillaries in cortex and ME**. High magnification images of capillaries immunostained for CD31 (white) show morphology of cortex and ME capillaries.

Supplementary Video 3 **Morphology of ECs in cortex and ME**. Imaris 3D reconstruction of single ECs (red) in cortex and ME labeled with tdTomato in *Cdh5-*CreERT2:Ai14 mouse. Co-staining for EC marker CD31 (white).

Supplementary Video 4 **Morphology of astrocytes in cortex and ME**. Imaris 3D reconstruction of single astrocytes (red) and tanycytes (yellow, in ME) in cortex and ME labeled with Tomato in *Slc1a3*-CreERT2:Ai14 mouse. Co-staining for EC marker CD31 (white) and Imaris 3D reconstruction of single GFAP^+^ astrocyte (green) in ME labeled with GFP in *GFAP-*EGFP mouse. Co-staining for EC marker CD31 (white).

Supplementary Video 5 **Serial TEM blood vessel reconstructions**. 3D reconstruction of two blood vessel-pericyte interactions from a serial TEM dataset of the visual cortex, highlighting pericytes (blue), an endothelial cell (green) and the blood vessel lumen (red).

Supplementary Video 6 **Morphology of pericytes in cortex and ME**. Imaris 3D reconstruction of single pericytes (red) in cortex and ME labeled with Tomato in *Pdgfrb*-CreERT2:Ai14 mouse. Co-staining for EC marker CD31 (white).

## Data Availability

The scRNA-seq data and GeoMX spatial profiling generated during this study are available for download at the Gene Expression Omnibus (GEO) (accession GSE241206). The analyzed scRNA-seq dataset has been uploaded to the Single Cell Portal (singlecell.broadinstitute.org/single_cell/study/SCP2553).

## Code availability

The source code to run ligand–receptor analysis is available at GitHub (https://github.com/gulabneuro/scRNAseq-ligand_receptor).
