# Supplementary material for: Characteristics of blood–brain barrier heterogeneity between brain regions revealed by profiling vascular and perivascular cells
Source: Nat Neurosci. 2024 Aug 29;27(10):1892–903. doi: 10.1038/s41593-024-01743-y (PMC11452347; doi:10.1038/s41593-024-01743-y)

nature neuroscience

Supplementary information https://doi.org/10.1038/s41593-024-01743-y

Characteristics of blood–brain barrier heterogeneity between brain regions revealed by profiling vascular and perivascular cells

In the format provided by the authors and unedited

SUPPLEMENTARY TABLES

Supplementary Table 1. scRNAseq cell type- and subtype-enriched genes, pathway analysis, spatial transcriptomics data and ligand-receptor interaction scores.

Supplementary Table 2. Antibodies used in study

Antibody	Product Number	RRID	Dilution
Primary Antibodies			
Mouse monoclonal anti-alpha SMA-Cy3 (clone 1A4)	Sigma-Aldrich C6198	AB_476856	1:150
Rabbit polyclonal anti-Aquaporin 4	Millipore AB3594	AB_91530	1:200
Goat polyclonal anti-Basigin/EMMPRIN	R&D Systems AF772	AB_355588	1:50
Goat polyclonal anti-CD31	R&D Systems AF3628	AB_2161028	1:50
Mouse monoclonal anti-Claudin-5 AF488 (clone 4C3C2)	ThermoFisher 352588	AB_2532189	1:100
Rabbit polyclonal anti-Collagen 1	Millipore AB765P	AB_92259	1:100
Goat polyclonal anti-Decorin	R&D Systems AF1060	AB_2090386	1:50
Rat monoclonal anti-Endomucin (clone V.7C7)	Santa Cruz sc-65495	AB_2100037	1:100
Rabbit monoclonal anti-ERG (clone EPR3864)	Abcam ab92513	AB_2630401	1:100
Rabbit monoclonal anti-ERG AF488 (clone EPR3864)	Abcam ab196374	AB_2889273	1:100
Goat polyclonal anti-Esm1/Endocan	R&D Systems AF1999	AB_2101810	1:50
Rabbit polyclonal anti-GFAP	Abcam ab7260	AB_305808	1:200
Chicken polyclonal anti-GFP	Aves GFP-1020	AB_10000240	1:200
Rabbit polyclonal anti-GFP	ThermoFisher A21311	AB_221477	1:150
Rabbit polyclonal anti-Glut1	Millipore 07-1401	AB_11212210	1:100
Rat monoclonal anti-Icam2/CD102 (clone 3C4(mIC2/4))	BD Biosciences 553326	AB_394784	1:100
Goat polyclonal anti-IGF1R1	R&D Systems AF-305	AB_354457	1:50
Rat monoclonal anti-Itga6 (clone GoH3)	R&D Systems MAB13501	AB_2128311	1:50
Rabbit polyclonal anti-KCC4/Slc12a7	Novus NBP1-85133	AB_11002763	1:500
Rabbit monoclonal anti-Lef1 (clone C12A5)	Cell Signaling 2230	AB_823558	1:100
Rabbit polyclonal anti-Mfsd2a	This study, J9590	NA	1:100
Goat polyclonal anti-PDGFRb	R&D Systems AF1042	AB_2162633	1:50
Rat monoclonal anti-Plvap (clone MECA32)	BD Biosciences 553849	AB_395086	1:100
Rabbit polyclonal anti-RFP	Rockland 600-401-379	AB_2209751	1:150
Goat polyclonal anti-Spock2	R&D Systems AF-493	AB_10717835	1:50

Goat polyclonal anti-VEGF	R&D Systems AF-493	AB_354506	1:50
Rat monoclonal anti-VEGFR2/Flk-1 (clone Avas 12α1)	BD Biosciences 555307	AB_395720	1:100
Chicken polyclonal anti-Vimentin	Millipore AB5733	AB_11212377	1:200
Secondary Antibodies			
donkey polyclonal anti-goat AF488	Jackson Immuno Research 705-545-147	AB_2336933	1:250, 1:300
donkey polyclonal anti-rabbit AF488	Jackson Immuno Research 711-545-152	AB_2313584	1:250
donkey polyclonal anti-rat AF488	Jackson Immuno Research 712-545-153	AB_2340684	1:250
donkey polyclonal anti-chicken AF488	Jackson Immuno Research 703-545-155	AB_2340375	1:250
donkey polyclonal anti-goat Cy3	Jackson Immuno Research 705-165-147	AB_2307351	1:250
donkey polyclonal anti-rabbit Cy3	Jackson Immuno Research 711-165-152	AB_2307443	1:250
donkey polyclonal anti-rat Cy3	Jackson Immuno Research 712-165-153	AB_2340667	1:250
donkey polyclonal anti-chicken Cy3	Jackson Immuno Research 703-165-155	AB_2340363	1:250
donkey polyclonal anti-goat AF647	Jackson Immuno Research 705-605-147	AB_2340437	1:250
donkey polyclonal anti-rabbit AF647	Jackson Immuno Research 711-605-152	AB_2492288	1:250, 1:300
donkey polyclonal anti-rat AF647	Jackson Immuno Research 712-605-153	AB_2340694	1:250
donkey polyclonal anti-chicken AF647	Jackson Immuno Research 703-605-155	AB_2340379	1:250
GeoMX Antibodies			
rabbit polyclonal anti-GFP-Alexa 488	Invitrogen A-21311	AB_221477	1:100
rabbit monoclonal anti-Desmin-Alexa 594 (clone Y66)	Abcam ab203419	AB_2943480	1:200
goat polyclonal anti-CD31-Alexa647	R&D Systems AF3628	AB_2161028	1:100

Supplementary Table 3. Marker genes used to classify cell types by scRNAseq

Cell Type	Marker Genes
Neurons	*Nrgn, Tubb3, Syt1, Snap25, Camk2b, Thy1*
Oligodendrocytes	*Cldn11, Mog, Mbp, Plp1, Mobp, Opalin, Olig1*
Oligodendrocyte precursor cells (OPCs)	*Olig1, Olig2, Pdgfra, Gpr17, Cspg4*
Astrocytes	*Aqp4, Aldoc, Slc1a2, Agt, Gja1, Atp1a2, Glul, Vegfa*
Tanycytes	*Gpr50, Rax, Crym, Vegfa, Vim, S100a6*
ECs	*Cdh5, Flt1, Ptprb, Tek*
Microglia	*P2ry12, C1qa, C1qb, Csf1r, Cx3cr1, Itgam, Tmem119, P2ry13*
Mural cells	*Rgs5, Notch3, Pdgfrb, Cspg4, Vtn, Acta2, Kcnj8, Abcc9, Myh11*
PVMs	*Cd74, Lyve1, Mrc1, Ptprc, H2.Eb1, H2.Ab1, Adgre1*
Pars tuberalis cells	*Apoc3, Chga, Cga, Chgb, Cck, Timeless, Tshb, Cyp2f2*
Fibroblasts	*Dcn, Col1a1, Col1a2, Col3a1, Lum, Pdgfra*
Ependymal cells	*Ccdc153, Rarres2, Vim, Tmem212, Foxj1*
T cells	*Ccl5, Ptprc, Trbc2, Cd52, Il2rb*
B cells	*Igkc*, *Cd79a*, *Ly6d*, *H2-DMb2*, and *Ighm*

SUPPLEMENTARY VIDEOS

Supplementary Video 1. Sulfo-NHS-Biotin leakage in cortex and ME

Immunostaining for EC marker CD31 (white) and BBB leakage tracer (Sulfo-NHS-Biotin, magenta) showing no leakage of tracer in cortex and tracer leaking out of vessels in ME.

Supplementary Video 2. Morphology of capillaries in cortex and ME

High magnification images of capillaries immunostained for CD31 (white) show morphology of cortex and ME capillaries.

Supplementary Video 3. Morphology of ECs in cortex and ME

Imaris 3D reconstruction of single ECs (red) in cortex and ME labeled with tdTomato in *Cdh5*-CreERT2: Ai14 mouse. Co-staining for EC marker CD31 (white).

Supplementary Video 4. Morphology of astrocytes in cortex and ME

Imaris 3D reconstruction of single astrocytes (red) and tanycytes (yellow, in ME) in cortex and ME labeled with Tomato in *Slc1a3*-CreERT2:Ai14 mouse. Co-staining for EC marker CD31 (white). And Imaris 3D reconstruction of single GFAP+ astrocyte (green) in ME labeled with GFP in *GFAP*-EGFP mouse. Co-staining for EC marker CD31 (white).

Supplementary Video 5. Serial TEM blood vessel reconstructions

3D reconstruction of two blood vessel-pericyte interactions from a serial TEM dataset of the visual cortex, highlighting pericytes (blue), an endothelial cell (green) and the blood vessel lumen (red).

Supplementary Video 6. Morphology of pericytes in cortex and ME

Imaris 3D reconstruction of single pericytes (red) in cortex and ME labeled with Tomato in *Pdgfrb*-CreERT2:Ai14 mouse. Co-staining for EC marker CD31 (white).

SUPPLEMENTARY FIGURES

Supplementary Fig. 1. Immune and oligodendrocyte cell subtypes revealed by single-cell RNA sequencing

Subclustering analyses of (a) immune and (b) oligodendrocyte cell types. In each panel there is (i) a UMAP projection of cell type subclusters, (ii) a UMAP projection of cell type subclusters colored by sample region, (iii) a bar graph showing the distribution of each subcluster within each sample region, (iv) a bar graph showing the distribution of each subcluster within each experimental replicate, and (v) a heatmap illustrating the top 5 genes differentiating each subcluster. Differentially expressed genes were determined by two-sided Wilcoxon test (min.pct=0.25) in Seurat comparing each subcluster to all other subclusters of each cell class. Replicates 12-15 contain samples from the ME only.

Supplementary Fig. 2. Mural and pars tuberalis cell subtypes revealed by single-cell RNA sequencing

Subclustering analyses of (a) mural and (b) pars tuberalis cell types. In each panel there is (i) a UMAP projection of cell type subclusters, (ii) a UMAP projection of cell type subclusters colored by sample region, (iii) a bar graph showing the distribution of each subcluster within each sample region, (iv) a bar graph showing the distribution of each subcluster within each experimental replicate, and (v) a heatmap illustrating the top 5 genes differentiating each subcluster. Differentially expressed genes were determined by two-sided Wilcoxon test (min.pct=0.25) in Seurat comparing each subcluster to all other subclusters of each cell class. Replicates 12-15 contain samples from the ME only.

Supplementary Fig. 3. Neuronal, tanycyte and ependymal cell subtypes revealed by single-cell RNA sequencing

Subclustering analyses of (a) neuron and (b) tanycyte and ependymal cell types. In each panel there is (i) a UMAP projection of cell type subclusters, (ii) a UMAP projection of cell type subclusters colored by sample region, (iii) a bar graph showing the distribution of each subcluster within each sample region, (iv) a bar graph showing the distribution of each subcluster within each experimental replicate, and (v) a heatmap illustrating the top 5 genes differentiating each subcluster. Differentially expressed genes were determined by two-sided Wilcoxon test (min.pct=0.25) in Seurat comparing each subcluster to all other subclusters of each cell class. Replicates 12-15 contain samples from the ME only.

Supplementary Fig. 1

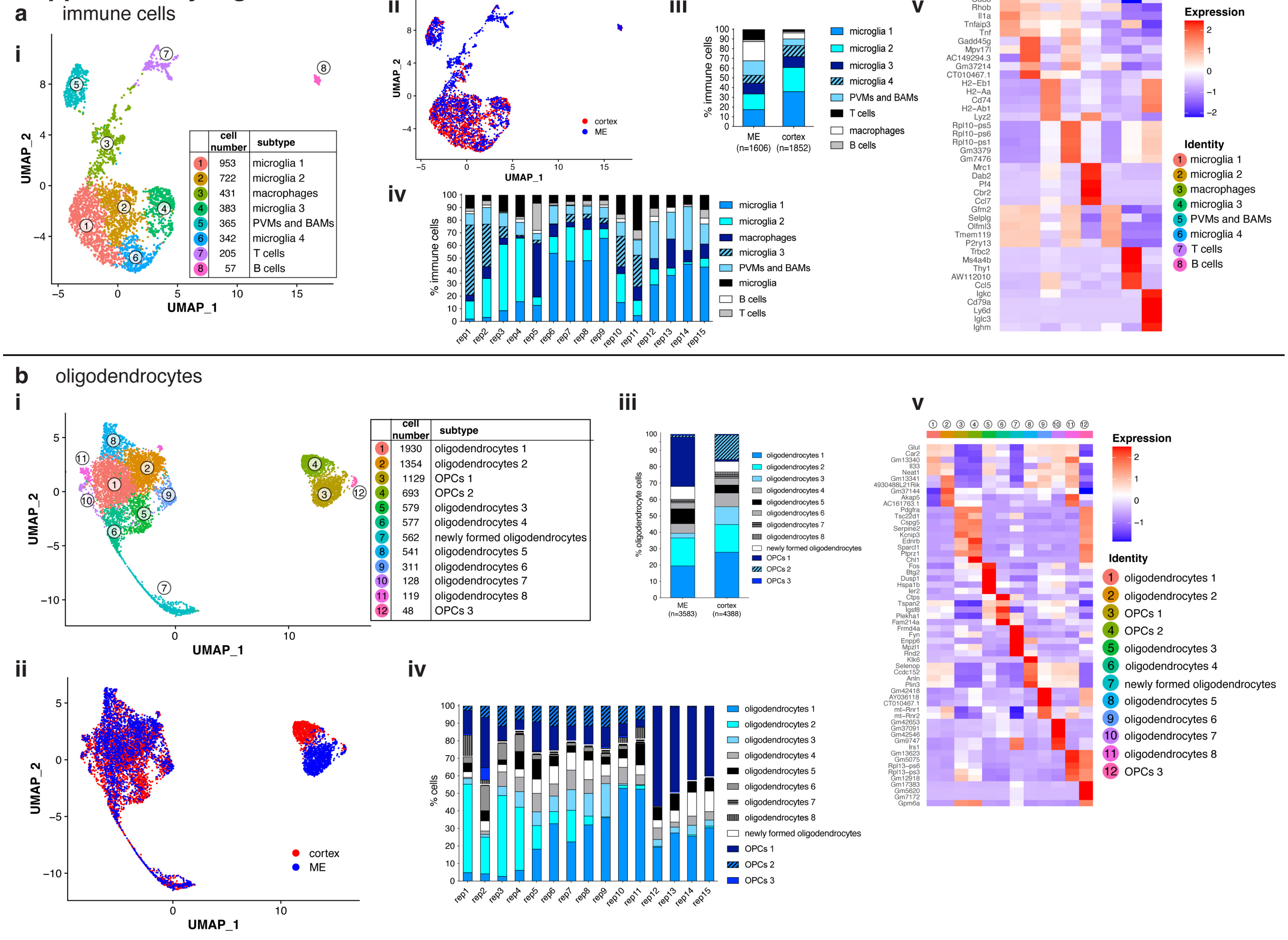

	cell number	subtype
1	953	microglia 1
2	722	microglia 2
3	431	macrophages
4	383	microglia 3
5	365	PVMs and BAMs
6	342	microglia 4
7	205	T cells
8	57	B cells

	cell number	subtype
1	1930	oligodendrocytes 1
2	1354	oligodendrocytes 2
3	1129	OPCs 1
4	693	OPCs 2
5	579	oligodendrocytes 3
6	577	oligodendrocytes 4
7	562	newly formed oligodendrocytes
8	541	oligodendrocytes 5
9	311	oligodendrocytes 6
10	128	oligodendrocytes 7
11	119	oligodendrocytes 8
12	48	OPCs 3

Supplementary Fig. 2

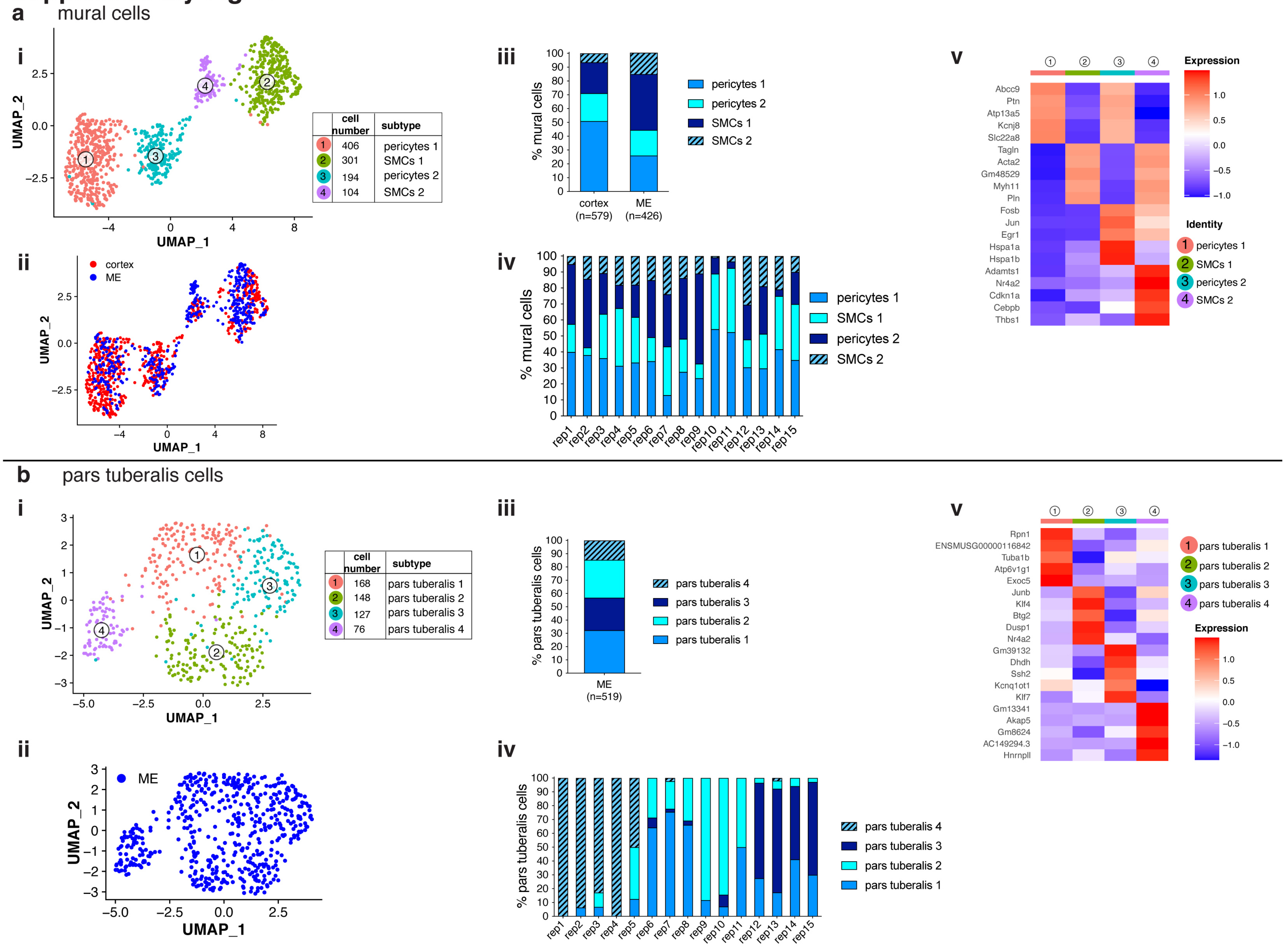

Supplementary Fig. 3

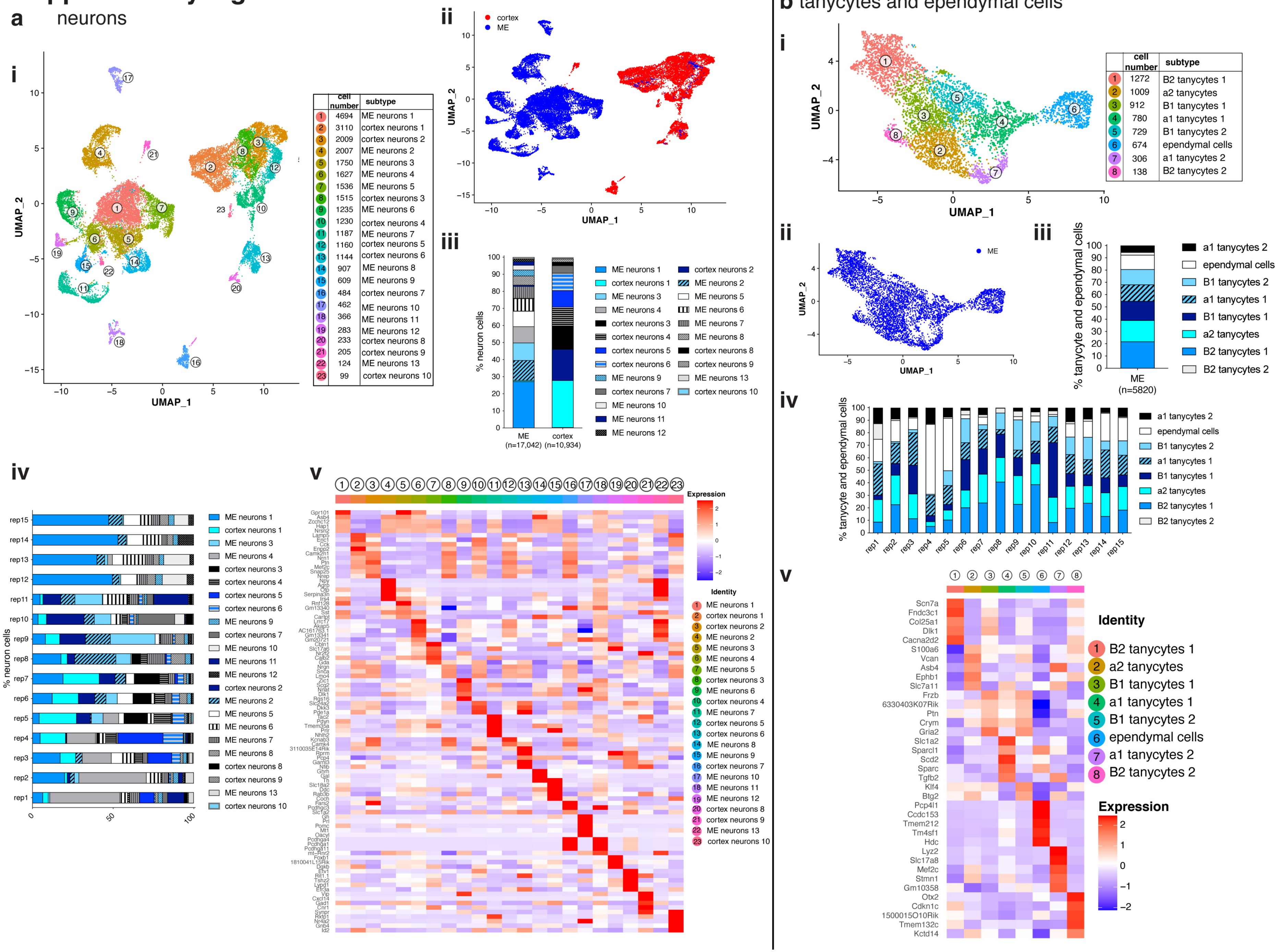

Supplement: Supplementary file 1 — Supplementary Tables 1–3, Supplementary Videos 1–6, Supplementary Figs. 1–3 [file 41593_2024_1743_MOESM1_ESM.pdf]
