# Supplementary material for: Characteristics of blood–brain barrier heterogeneity between brain regions revealed by profiling vascular and perivascular cells
Source: Nat Neurosci. 2024 Aug 29;27(10):1892–903. doi: 10.1038/s41593-024-01743-y (PMC11452347; doi:10.1038/s41593-024-01743-y)
Supplement: Supplementary file 2 — Reporting Summary [file 41593_2024_1743_MOESM2_ESM.pdf]

nature portfolio

Corresponding author(s): Chenghua Gu

Last updated by author(s): Jul 19, 2024

Reporting Summary

Nature Portfolio wishes to improve the reproducibility of the work that we publish. This form provides structure for consistency and transparency in reporting. For further information on Nature Portfolio policies, see our Editorial Policies and the Editorial Policy Checklist.

Statistics

For all statistical analyses, confirm that the following items are present in the figure legend, table legend, main text, or Methods section.

n/a	Confirmed	
☐	☒	The exact sample size (*n*) for each experimental group/condition, given as a discrete number and unit of measurement
☐	☒	A statement on whether measurements were taken from distinct samples or whether the same sample was measured repeatedly
☐	☒	The statistical test(s) used AND whether they are one- or two-sided<br>*Only common tests should be described solely by name; describe more complex techniques in the Methods section.*
☒	☐	A description of all covariates tested
☐	☒	A description of any assumptions or corrections, such as tests of normality and adjustment for multiple comparisons
☐	☒	A full description of the statistical parameters including central tendency (e.g. means) or other basic estimates (e.g. regression coefficient) AND variation (e.g. standard deviation) or associated estimates of uncertainty (e.g. confidence intervals)
☐	☒	For null hypothesis testing, the test statistic (e.g. *F*, *t*, *r*) with confidence intervals, effect sizes, degrees of freedom and *P* value noted<br>*Give P values as exact values whenever suitable.*
☒	☐	For Bayesian analysis, information on the choice of priors and Markov chain Monte Carlo settings
☒	☐	For hierarchical and complex designs, identification of the appropriate level for tests and full reporting of outcomes
☒	☐	Estimates of effect sizes (e.g. Cohen's *d*, Pearson's *r*), indicating how they were calculated

Our web collection on statistics for biologists contains articles on many of the points above.

Software and code

Policy information about availability of computer code

Data collection

The following software's were used to collect the data in this study:
- LAS X 3.0.16120.2 for Leica SP8 Confocal Imaging
- OlyVIA Ver.2.9.1 for VS120 Virtual Slide Microscope
- AMT_V700 for electron microscopy imaging

Data analysis

Imaris 9, Oxford Instruments, https://imaris.oxinst.com/
TrakEM2, Cardona et al., 2012, https://imagej.net/TrakEM2
FIJI (2.1.0) Schindelin et al., 2012, https://fiji.sc
Blender (2.90.0), Blender Institute, https://www.blender.org
bcbio-nextgen (1.2.8) https://bcbio-nextgen.readthedocs.io/en/latest/contents/single_cell.html
Python (3.8.3) https://www.python.org
scrublet (0.2.3), Wolock et al., 2019. https://github.com/swolock/scrublet
emptyDrops, Lun et al., 2019 DropletUtils package (1.8.0): https://bioconductor.org/packages/release/bioc/html/DropletUtils.html
SoupX (1.5.0), Young and Behjati, 2020. https://github.com/constantAmateur/SoupX
scProportionTest (0.0.0.9000), Miller et al., 2021, https://github.com/rpolicastro/scProportionTest
R version 4.0.2, R Core Team, 2020, https://www.r-project.org
R version 4.1.1, R Core Team, 2021, https://www.r-project.org
R version 4.1.3, R Core Team, 2022, https://ww.r-project.org
RStudio (2023.09.1+494) , RStudio Team, 2016, http://www.rstudio.com
Seurat version 4, Macosko et al., 2015; Butler et al., 2018; Hao, Hao, et al., 2021 https://satijalab.org/seurat/
bc3net R package (1.0.4), de Matos Simoes et al., 2012. https://cran.r-project.org/web/packages/bc3net/index.html

pheatmap R package(1.0.12) https://cran.r-project.org/web/packages/pheatmap/index.html
ggvenn R package (0.1.10) https://cran.r-project.org/web/packages/ggvenn/index.html
EnhancedVolcano R package (1.6.0), Blighe et al., 2024. https://bioconductor.org/packages/release/bioc/html/EnhancedVolcano.html
GSVA R package (1.38.2), Hänzelmann et al., 2013. https://bioconductor.org/packages/release/bioc/html/GSVA.html
harmony R package(1.0.3) Korsunsky et al., 2019, https://github.com/immunogenomics/harmony
UpsetR R package (1.4.0),Conway et al., 2017. https://cran.r-project.org/web/packages/UpSetR/index.html
Prism 8, GraphPad, https://www.graphpad.com/scientific-software/prism/
Interaction Score algorithm, Kumar et al., 2018, https://github.com/mkumar45/syngeneic_scRNAseq; this paper, https://github.com/gulabneuro/scRNAseq
GeomxTools R package (3.1.1), Ortogero et al., 2023 https://www.bioconductor.org/packages/release/bioc/html/GeomxTools.html
CellChat R package (1.6.0), Jin et al., 2021 https://github.com/sqjin/CellChat

For manuscripts utilizing custom algorithms or software that are central to the research but not yet described in published literature, software must be made available to editors and reviewers. We strongly encourage code deposition in a community repository (e.g. GitHub). See the Nature Portfolio guidelines for submitting code & software for further information.

Data

Policy information about availability of data

All manuscripts must include a data availability statement. This statement should provide the following information, where applicable:

- Accession codes, unique identifiers, or web links for publicly available datasets
- A description of any restrictions on data availability
- For clinical datasets or third party data, please ensure that the statement adheres to our policy

The sequencing data (single cell and GeoMX) generated during this study are available for download at GEO (accession GSE241206).
The scRNAseq database can be accessed interactively at https://singlecell.broadinstitute.org/single_cell/study/SCP2553.
The source code to run ligand-receptor analysis is available at https://github.com/gulabneuro/scRNAseq-ligand_receptor/.

The following publicly available databases were used for analysis:
MSigDB (https://www.gsea-msigdb.org/gsea/msigdb)
UniProt (https://www.uniprot.org/)
STRING (https://string-db.org/)

The following studies were used for comparative scRNAseq analysis:
Chen et al., 2020 ,PMID: 31915267
Dani et al. 2021, PMID: 33932339
Elmentaite et al., 2021, PMID: 34497389
He et al., 2021, PMID: 33837218
Kalucka et al. 2020, PMID: 32059779
Saunders et al. 2018, PMID: 30096299
Travaglini et al., 2020, PMID: 332089466Feng et al. 2019 PMID: 31850371
Yang et al. 2022, PMID: 35165441
Wang et al. 2019, PMID: 30932813
Zeisel et al. 2018, PMID: 30096314

Research involving human participants, their data, or biological material

Policy information about studies with human participants or human data. See also policy information about sex, gender (identity/presentation), and sexual orientation and race, ethnicity and racism.

Reporting on sex and gender	NA
Reporting on race, ethnicity, or other socially relevant groupings	NA
Population characteristics	NA
Recruitment	NA
Ethics oversight	NA

Note that full information on the approval of the study protocol must also be provided in the manuscript.

Field-specific reporting

Please select the one below that is the best fit for your research. If you are not sure, read the appropriate sections before making your selection.

☒ Life sciences ☐ Behavioural & social sciences ☐ Ecological, evolutionary & environmental sciences

For a reference copy of the document with all sections, see nature.com/documents/nr-reporting-summary-flat.pdf

Life sciences study design

All studies must disclose on these points even when the disclosure is negative.

Sample size	For confocal and electron microscopy data, we performed preliminary experiments to identify the variation. We then perform a power test to identify appropriate sample sizes of images per mouse. Based on previous experience with similar studies, the sample sizes were sufficient. For transcriptomic experiments, sample sizes were chosen based on the yield of high quality vascular cells. For scRNAseq, we aimed to profile at least 100 cells per cluster from each region of our cell types of interest. For GeoMX, we based our sample size on reproducible clustering of samples from multiple animals on separate experiment days both by sample region and enriched cell type.
Data exclusions	Images were only excluded when the quality of the images was too poor for data analysis.
Replication	All representative stainings and TEM images have been performed in equal to or more than 3 mice in at least 3 independent experiments. inDrops scRNAseq samples were collected on 15 separate days, and sequencing libraries were generated over 9 days to minimize variation due to library preparation. For GeoMX DSP, samples from 8 animals of both sexes were profiled over 3 separate days. In all cases, attempts at replication were successful.
Randomization	Mice were randomized based on their genotypes and allocated randomly into their respective genotype group.
Blinding	Acquisition, collection and analysis of the experiments were performed all blinded to the genotypes. Only after the data was completely analyzed were the genotypes unblinded.

Reporting for specific materials, systems and methods

We require information from authors about some types of materials, experimental systems and methods used in many studies. Here, indicate whether each material, system or method listed is relevant to your study. If you are not sure if a list item applies to your research, read the appropriate section before selecting a response.

Materials & experimental systems

n/a	Involved in the study	
☐	☒	Antibodies
☒	☐	Eukaryotic cell lines
☒	☐	Palaeontology and archaeology
☐	☒	Animals and other organisms
☒	☐	Clinical data
☒	☐	Dual use research of concern
☒	☐	Plants

Methods

n/a	Involved in the study	
☒	☐	ChIP-seq
☒	☐	Flow cytometry
☒	☐	MRI-based neuroimaging

Antibodies

Antibodies used

Mouse monoclonal anti-alpha SMA-Cy3 (clone 1A4), Sigma-Aldrich C6198; RRID: AB_476856; 1:150
Rabbit polyclonal anti-Aquaporin 4, Millipore AB3594; RRID: AB_91530; 1:200
Goat polyclonal anti-Basigin/EMMPRIN, R&D Systems AF772; RRID: AB_355588; 1:50
Goat polyclonal anti-CD31, R&D Systems AF3628; RRID: AB_2161028; 1:50
Mouse monoclonal anti-Claudin-5 AF488 (clone 4C3C2), Thermo Fisher 352588; RRID: AB_2532189; 1:100
Rabbit polyclonal anti-Collagen 1, Millipore AB765P; RRID: AB_92259; 1:100
Goat polyclonal anti-Decorin, R&D Systems AF1060; RRID: AB_2090386; 1:50
Rat monoclonal anti-Endomucin (clone V.7C7) ,Santa Cruz sc-65495; RRID: AB_2100037; 1:100
Rabbit monoclonal anti-ERG (clone EPR3864), Abcam ab92513; RRID: AB_2630401; 1:100
Rabbit monoclonal anti-ERG AF488 (clone EPR3864), Abcam ab196374; RRID: AB_2889273; 1:100
Goat polyclonal anti-Esm1/Endocan, R&D Systems AF1999; RRID: AB_2101810; 1:50
Rabbit polyclonal anti-GFAP, Abcam ab7260; RRID: AB_305808; 1:200
Chicken polyclonal anti-GFP, Aves GFP-1020; RRID: AB_10000240; 1:200
Rabbit polyclonal anti-GFP, Thermo Fisher A21311; RRID: AB_221477; 1:150
Rabbit polyclonal anti-Glut1, Millipore 07-1401; RRID: AB_11212210; 1:100
Rat monoclonal anti-Icam2/CD102 (clone 3C4 (mIC2/4)), BD Biosciences 553326; RRID: AB_394784; 1:100
Goat polyclonal anti-IGF1R1, R&D Systems AF-305; RRID: AB_354457; 1:50
Rat monoclonal anti-Itga6 (clone GoH3), R&D Systems MAB13501; RRID: AB_2128311; 1:50
Rabbit polyclonal anti-KCC4, Novus, NBP1-85133; RRID: RRID:AB_11002763; 1:500
Rabbit monoclonal anti-LEF1 (clone C12A5), Cell Signaling 2230; RRID: AB_823558; 1:100
Rabbit polyclonal anti-Mfsd2a, This paper J9590; RRID: NA; 1:100
Goat polyclonal anti-PDGFRb, R&D Systems AF1042; RRID: AB_2162633; 1:50
Rat monoclonal anti-Plvap (clone MECA32), BD Biosciences 553849; RRID: AB_395086; 1:100
Goat polyclonal anti-Spock2, R&D Systems AF2328; RRID: AB_10717835; 1:50
Rabbit polyclonal anti-RFP, Rockland 600-401-379; RRID: AB_2209751; 1:150
Goat polyclonal anti-VEGF, R&D Systems AF-493; RRID: AB_354506; 1:50
Rat monoclonal anti-VEGFR2/Flk-1 (clone Avas 12a1), BD Biosciences 555307; RRID: AB_395720; 1:100

Chicken polyclonal anti-Vimentin, Millipore AB5733; RRID: AB_11212377; 1:200
Rabbit polyclonal anti-GFP-Alexa488, Invitrogen A-21311; RRID: AB_221477; 1:100
Rabbit monoclonal anti-Desmin-Alexa594 (clone Y66), Abcam Y66, ab203419; RRID: AB_2943480; 1:200
Goat polyclonal anti-CD31-Alexa647, R&D Systems, AF3628; RRID: AB_2161028; 1:100
donkey polyclonal anti-goat AF488, Jackson Immuno Research 705-545-147; RRID: AB_2336933; 1:250, 1:300
donkey polyclonal anti-rabbit AF488, Jackson Immuno Research 711-545-152; RRID: AB_2313584; 1:250
donkey polyclonal anti-rat AF488, Jackson Immuno Research 712-545-153; RRID: AB_2340684; 1:250
donkey polyclonal anti-chicken AF488, Jackson Immuno Research 703-545-155; RRID: AB_2340375; 1:250
donkey polyclonal anti-goat Cy3, Jackson Immuno Research 705-165-147; RRID: AB_2307351; 1:250
donkey polyclonal anti-rabbit Cy3, Jackson Immuno Research 711-165-152; RRID: AB_2307443; 1:250
donkey polyclonal anti-rat Cy3, Jackson Immuno Research 712-165-153; RRID: AB_2340667;1:250
donkey polyclonal anti-chicken Cy3, Jackson Immuno Research 703-165-155; RRID: AB_2340363; 1:250
donkey polyclonal anti-goat AF647, Jackson Immuno Research 705-605-147; RRID: AB_2340437; 1:250
donkey polyclonal anti-rabbit AF647, Jackson Immuno Research 711-605-152; RRID: AB_2492288; 1:250, 1:300
donkey polyclonal anti-rat AF647, Jackson Immuno Research 712-605-153; RRID: AB_2340694; 1:250
donkey polyclonal anti-chicken AF647, Jackson Immuno Research 703-605-155; RRID: AB_2340379; 1:250

Validation

anti-alpha SMA-Cy3 is valid because staining was specifically localized to smooth muscle cells on arteries.
anti-Aquaporin 4 is valid because staining was specifically localized to astrocyte endfeet and used in many publications.
anti-Basigin/EMMPRIN is valid because the staining is consistent with our single cell RNA sequencing data, it is used in many publications and tested by the manufacturer.
anti-CD31 is valid because the staining is consistent with the known expression of endothelial cells and extensively used in many publications.
anti-Claudin-5 is valid because the staining is consistent with the known expression of CNS endothelial cells and extensively used in many publications.
anti-Collagen 1 is valid because the staining is consistent with the known expression in fibroblasts, it is extensively used in many publications and tested by the manufacturer.
anti-Decorin is valid because the staining is consistent with known expression in fibroblasts, matching our single cell RNA sequencing data and testing by the manufacturer.
anti-Endomucin is valid because the staining is consistent with the known expression in endothelial cells and extensively used in many publications.
anti-ERG and anti-ERG A488 are valid because the staining is consistent with the known expression in endothelial cell nuclei and extensively used in many publications.
anti-Esm1/Endocan is valid because the staining is consistent with the known expression in endothelial cells and extensively used in many publications.
anti-GFAP is valid because the staining is consistent with endogenous GFAP expression and extensively used in many publications.
chicken anti-GFP is valid because the staining is consistent with expression in reporter mice and extensively used in many publications
rabbit anti-GFP is valid because the staining is consistent with expression in reporter mice and extensively used in many publications.
anti-Glut1 is valid because the staining is consistent with the known expression of CNS endothelial cells and extensively used in many publications.
anti-Icam2/CD102 is valid because the staining is consistent with the known expression of endothelial cells and manufacturer routinely test by flow cytometry.
anti-IGF1R1 is valid because the staining is consistent with our single cell RNA sequencing data and used in many publications.
anti-ITGA6 is valid because the staining is consistent with our single cell RNA sequencing data and used in many publications.
anti-KCC4 is valid because the staining is consistent with our spatial transcriptomics data and used in several publications.
anti-LEF1 is valid because the staining is consistent with its known expression pattern in brain endothelial cells and reporter mice and it is extensively used in many publications.
anti-Mfsd2a has been validated on Mfsd2a brain KO tissue see Extended Data Fig. 1 f
anti-PDGFRb is valid because the staining is consistent with the known expression in pericytes and extensively used in many publications.
anti-Plvap/Meca32 is valid because the staining is consistent with the known expression in ME endothelial cells and extensively used in many publications.
anti-Spock2 has been validated on Spock2 brain KO tissue see Extended Data Fig. 5 a
anti-RFP is valid because the staining is consistent with endogenous RFP expression and extensively used in many publications.
anti-VEGF is valid because the staining is consistent with scRNAseq data and used in many publications.
anti-VEGFR2/Flk-1 is valid because the staining is consistent with the known expression of CNS endothelial cells and extensively used in many publications.
anti-Vimentin is valid because the staining is consistent with the known expression in tanycytes and extensively used in many publications.
anti-GFP-Alexa488, anti-Desmin-Alexa594 and anti-CD31-Alexa647 staining is consistent with genetic reporters. All were also validated by NanoString for compatibility with theGeoMX platform.

Animals and other research organisms

Policy information about studies involving animals; ARRIVE guidelines recommended for reporting animal research, and Sex and Gender in Research

Laboratory animals

All animal experiments were approved by the Harvard University Institutional Animal Care and Use Committee (IACUC). Mice were maintained on a 12-hour light/12-hour dark cycle at 71 degrees Fahrenheit and 55% humidity. All mice used for analysis were 8 to 14 weeks old unless stated otherwise. Both male and female mice were used in all experiments unless otherwise indicated. The following mouse strains were used: wild type (C57BL/6N, Charles River Laboratories #027), Ai14 (JAX: 007914), Aldh1l1-EGFP (JAX: 026033), GFAP-GFP (JAX: 003257), Glast-CreER (JAX: 012586), TCF/LEF-GFP (JAX: 032577), Cdh5-CreERT2 (Wang et al., 2010),

	Slco1c1-CreERT2 (Ridder et al., 2011), Pdgfrb-CreERT2 (JAX: 029684), Pdgfra-H2B-EGFP (JAX: 007669), Mfsd2ako (MMRRC strain 032467-UCD), and ROSA26LSL-ER-HRP (JAX: 034746).
Wild animals	No wild animals were used in this study.
Reporting on sex	Male mice were used for scRNAseq experiments because the ME is involved in the secretion of hormones related to estrus. Male and female mice were used in all other experiments.
Field-collected samples	No field-collected samples were used in this study.
Ethics oversight	All mouse experiments followed institutional and US National Institute of Health (NIH) guidelines and were approved by the Harvard University Institutional Animal Care and Use Committee.

Note that full information on the approval of the study protocol must also be provided in the manuscript.
